# Current Advances in Nano-Based and Polymeric Stimuli-Responsive Drug Delivery Targeting the Ocular Microenvironment: A Review and Envisaged Future Perspectives

**DOI:** 10.3390/polym14173580

**Published:** 2022-08-30

**Authors:** Siphokazi B. K. Dludla, Leshasha T. Mashabela, Brian Ng’andwe, Pedzisai A. Makoni, Bwalya A. Witika

**Affiliations:** 1 Division of Pharmaceutics, Faculty of Pharmacy, Rhodes University, Makhanda 6140, South Africa; 2 Department of Pharmaceutical Sciences, School of Pharmacy, Sefako Makgatho Health Sciences University, Pretoria 0208, South Africa; 3 University Teaching Hospitals-Eye Hospital, Private Bag RW 1 X Ridgeway, Lusaka 10101, Zambia; 4 Division of Pharmacology, Faculty of Pharmacy, Rhodes University, Makhanda 6140, South Africa

**Keywords:** ocular drug delivery, stimuli-responsive drug delivery, nanotechnology, on-target drug delivery

## Abstract

Optimal vision remains one of the most essential elements of the sensory system continuously threatened by many ocular pathologies. Various pharmacological agents possess the potential to effectively treat these ophthalmic conditions; however, the use and efficacy of conventional ophthalmic formulations is hindered by ocular anatomical barriers. Recent novel designs of ophthalmic drug delivery systems (DDS) using nanotechnology show promising prospects, and ophthalmic formulations based on nanotechnology are currently being investigated due to their potential to bypass these barriers to ensure successful ocular drug delivery. More recently, stimuli-responsive nano drug carriers have gained more attention based on their great potential to effectively treat and alleviate many ocular diseases. The attraction is based on their biocompatibility and biodegradability, unique secondary conformations, varying functionalities, and, especially, the stimuli-enhanced therapeutic efficacy and reduced side effects. This review introduces the design and fabrication of stimuli-responsive nano drug carriers, including those that are responsive to endogenous stimuli, viz., pH, reduction, reactive oxygen species, adenosine triphosphate, and enzymes or exogenous stimuli such as light, magnetic field or temperature, which are biologically related or applicable in clinical settings. Furthermore, the paper discusses the applications and prospects of these stimuli-responsive nano drug carriers that are capable of overcoming the biological barriers of ocular disease alleviation and/or treatment for in vivo administration. There remains a great need to accelerate the development of stimuli-responsive nano drug carriers for clinical transition and applications in the treatment of ocular diseases and possible extrapolation to other topical applications such as ungual or otic drug delivery.

## 1. Introduction

Vision is one of the most essential elements of the sensory system that most people fear losing. However complex, the physiology of the various anatomical structures of the visual system allows for them to function together as a single unit to provide optimum visualization [1]. Although the eye has been identified as one of the organs that possess a pathologies capable of causing visual impairment and blindness. Many ocular diseases require the use pharmacological agents for effective management [2,3]. Various ophthalmic drugs can be used to treat ocular diseases; however, the delivery of these drugs remains a challenge due to the high degree of complexity of the ocular anatomy and physiology acting as a barrier hindering successful delivery of ocular drugs [4]. The cornea and the tear film have been identified as two of the major components of the visual system acting as barriers to attaining effective ocular drug delivery [5].

Various dosage forms exist for the delivery of ophthalmic drugs and are classified according to their physical forms i.e. suspensions, ointments, contact lenses, ocular implants, punctual plugs, and eyedrops. The market for ophthalmic products is largely occupied by topical formulations, with eyedrops accounting for ~90% of ophthalmic formulations currently commercially available [4,6,7]. These conventional topical drug formulations associate with many challenges when used to treat eye diseases, i.e., reduced corneal residence time leading to poor drug bioavailability [8]. The bioavailability of ocular drugs administered via the topical route is estimated to be 5%, and even lower drug amounts reach the posterior segment because of the blood retinal barrier (BRB) and blood ocular barriers (BOB) [4,7].

Following instillation, the ocular tear film mixes with the administered eye drops, resulting in the tear film turnover rate causing a reduction in the drug’s resident time on the corneal surface (~2–5 min) [8]. Failure by the cornea to successfully absorb administered drugs results in the subsequent absorption (~50%) of administered drug molecules by either the conjunctiva, which is more permeable than the cornea and has a larger surface area, or they get drained into the lacrimal sac, and get absorbed into the systemic blood circulation through the lacrimal system [8,9]. This unintended systemic absorption results in unwanted systemic adverse effects; moreover, the drug gets wasted since it fails to reach the intended site of action, hence therapy failure results [8].

The use of alternative routes of administration using invasive techniques such as the intravitreal (IVT) route to administer ocular drugs, allows for targeted drug delivery to the posterior segment. However, it is associated with undesirable outcomes i.e., increased risk of developing endophthalmitis, contact cataracts, retinal detachment, and glaucoma. The less invasive periocular route does not cause any unwanted adverse effects seen with IVT, but it suffers from decreased drug bioavailability at the targeted site due to anatomical barriers. Similar bioavailability issues have been identified with the use of systemic routes i.e., using intravenous administration results in only 1–2% of the drug reaching targeted ocular tissue [7,10]. Considering these facts, the topical route of administration remains the most preferable route for ophthalmic drug delivery because it is safe, efficient, allows for self-administration (unlike invasive techniques requiring trained specialists), and is non-invasive [11].

Attempts have been made to improve the bioavailability of topically administered ophthalmic drugs, however, they have not proven successful [4]. One of the approaches included the formulation of eyedrops using high drug concentrations, which resulted in undesirable ocular tissue irritation. Furthermore, the subsequent systemic absorption by the lacrimal system exacerbated systemic adverse effects already seen with the use of conventional eyedrops. Other attempts included the incorporation of cyclodextrins in formulations to enhance the permeability of drugs, the inclusion of efflux pump inhibitors, increasing the viscosity of ophthalmic formulations i.e., ophthalmic ointments, and increasing the frequency of daily dosing. The use of efflux pump inhibitors was suspected to cause unwanted adverse effects with prolonged use, whilst increasing the viscosity interfered with light transmission which caused temporary blurring of vision, and repeated daily dosing led to poor patient compliance and adherence which all resulted in therapy failure [4,9,12].

More recently, ophthalmic formulations based on nanotechnology are being investigated, using nanocarriers as DDS, including polymeric nanoparticles and nanomicelles for targeted drug delivery [6,10,13]. Other promising novel approaches include the use of liposomes, niosomes, nanoemulsions, and nanosuspensions [10,13,14].

**Figure 1:**
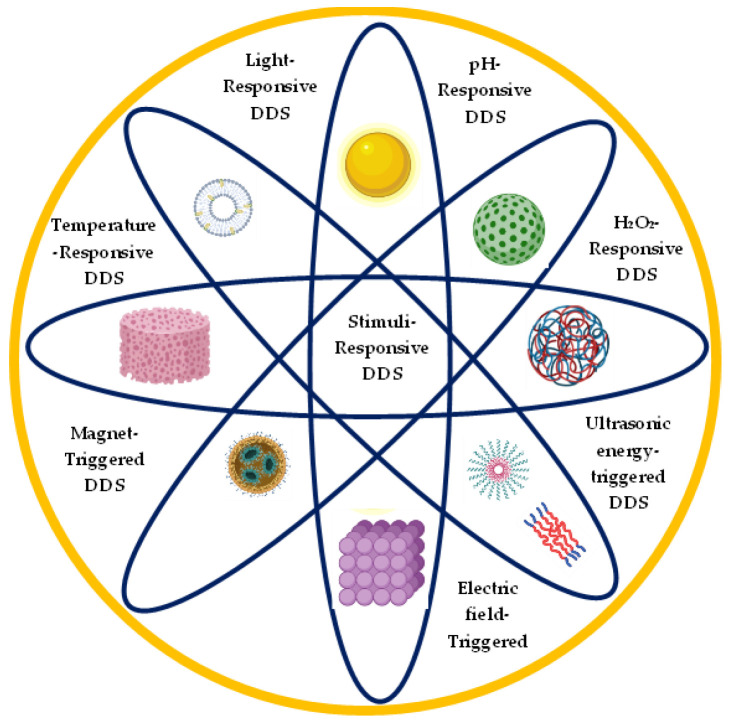
Schematic diagram of different stimuli-responsive ocular drug delivery systems and different nanomaterials used in ocular drug delivery. Adapted from [15]. 2022, Elsevier Under Creative Commons license.

Over the past years, stimuli-responsive delivery systems have gained more attention as they show promising prospects for the delivery of drugs in a controlled manner. Stimuli-responsive drug release means that drug release can be achieved in response to a patient’s physiological condition, including temperature, light, and pH. Different stimuli-responsive delivery systems and nanomaterials are currently being explored for application in ocular drug delivery as depicted in Figure 1 [15].

This review introduces the design and fabrication of stimuli-responsive nano drug carriers that are responsive to endogenous stimuli viz., pH, reduction, reactive oxygen species, adenosine triphosphate and enzyme or exogenous stimuli like light, magnetic field or temperature which are biologically related or applicable in clinical settings. Furthermore, the paper discusses the applications and prospects of these stimuli-responsive nano drug carriers that are capable of overcoming the biological barriers of ocular disease alleviation and/or treatment for in vivo administration.

## 2. Ocular Anatomy and Physiology

**Figure 2:**
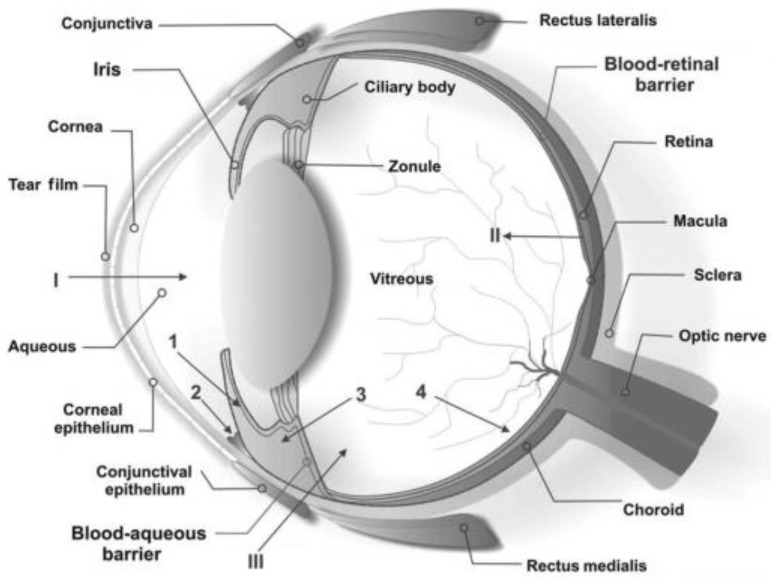
Schematic representation of the various anatomical structures of the eye and the physiological protective mechanisms, including tear turnover (tear film), lowly permeable cornea, and the blood retinal barrier. The primary physiologic blockage against instilled ocular drugs is the tear film. Reproduced from [16] with permission of John Wiley and Sons.

The eye is anatomically and physiologically a very specialized organ with an ultimate function of focusing and processing of light through a nerve network for onward interpretation by the brain [16]. The anatomy and protective mechanisms of the eye, depicted in Figure 2, presents a two-fold challenge for drug delivery.

The eyeball makes up about one fifth of the orbit. It is composed of two segments, the larger posterior segment and the smaller anterior segment. The anterior segment is further divided into the anterior and posterior chamber. The conjunctiva, sclera, cornea, anterior chamber, iris, ciliary body, posterior chamber, and lens, make up the anterior chamber. The posterior chamber consists of the vitreous humour, the retina, optic nerve and the choroid [17].

Barriers do exist that protect the eye from foreign substances. Firstly, that tear turnover and low permeability of the cornea might result in low intra-ocular bioavailability of topical drugs and secondly, that the blood-aqueous and blood-retina barrier might make systemic drugs less effective. Many of these barriers are unique to ocular anatomy and physiology and thus pose a challenge to drug delivery. Further, the barriers are specific to the route of delivery of the drug. Understanding the anatomy and physiology of the eye is thus cardinal in drug development [18].

### 2.1. Topical Ocular Medication

Topical medication may be applied as eye drops, gels or ointments. These will mostly help in treating anterior segment ocular conditions such as uveitis and conjunctivitis. The tissues affected by topical medications are the cornea, conjunctiva, sclera and the uvea (i.e. iris and ciliary body).

The transparent cornea together with the opaque sclera make up the outermost layer of the eyeball. They offer a physiological and anatomical barrier to any topical substance applied, and for drugs this may affect the bioavailability. The thickness of the cornea varies, with the center being thinner ~551 to 565 μm than the periphery ~612 to 1000 μm. There are 5 layers within the cornea which include the epithelium, bowman’s layer, stroma, Descemet membrane, and the endothelium which offer several challenges for drug penetration into the eye [16].

Several barriers do exist to prevent trans-corneal entry of substances into the eye. The epithelium has tight junctions that form a barrier to hydrophilic molecules. Characteristically, the epithelium and stroma limit the permeation of substances through the cornea into the eye by forming lipophilic and hydrophilic barriers respectively. Further, the cornea has micropores measuring approximately 2 nm and has a relatively low pore density than the conjunctiva, thus smaller particles have to permeate via the transcellular path [19].

The pre-corneal factors such as the tear film, tear drainage, blinking, induced lacrimation and tear turnover are key challenges that drug formulation scientists have to overcome in developing topical medications [20].

The tear film is a trilaminar film on the anterior surface of the eye composed of an outer lipid layer, middle aqueous layer, and an inner mucin layer. It has a significant role in eliminating unwanted substances including germs and toxins. The tear volume is approximately 7–30 µL with the turnover time of 0.5–2.2 µL/min. The average blink rate of individuals is 15 times/min, which reduces the maximum volume of tears in the conjunctival sac to approximately 6–7 µL [21]. For topical medications, this might mean that only 20% (50 µL) of the formulation will be retained in the conjunctival sac whilst the rest is lost to overflow. Thus, whilst there is less concern with regard to systemic loss of topical drugs, these factors combined lead to significant loss.

The sclera is relatively avascular with most of the vascularity being within the episclera. The tenon capsule is a sub-conjunctival tissue that is attached to the sclera. The sclera is thickest (~1 mm) posteriorly and thinnest (~0.3 mm) just before the insertion of the extraocular muscles [22]. The sclera is hydrated and has large collagen fibrils arranged haphazardly; therefore, it is opaque and white rather than clear. There is better permeability of substances through the sclera although intraocular delivery might be compromised by the vascular beds in the episcleral [23].

The uvea is the vascular coating of the eyeball and consists of the iris, ciliary body, and the choroid. The ciliary body has tight junctions between the inner non pigmented epithelium which prevent exudate from the fenestrated ciliary capillaries reaching the aqueous humour [22]. This thus creates a blood aqueous barrier. It should, however, be noted that there are no tight junctions between the pigmented ciliary epithelium. In the iris, there is no protective epithelial barrier thus the non-fenestrated vessels offer the blood aqueous barrier [16].

The pigmented epithelium of the iris and ciliary body contain melanin which can bind to substances such as drugs. This might lead to prolonged but reduced effects of a drug due to slow release. Thus, topical delivery though noninvasive fails to deliver significant therapeutic effects in the posterior segment [24].

### 2.2. Systemic (Oral and Parenteral) Ocular Drug Administration

Drugs administered orally or parenterally have to overcome the blood ocular barriers to offer meaningful therapeutic effects in the eye. The blood aqueous humour barrier is an important barrier for drug delivery into the anterior segment. It consists of the cells of the endothelium of iris/ciliary blood vessels and the non-pigmented ciliary epithelium that have tight junctions which prevent entry of substances into the eye [20].

The aqueous humour is mostly (70%) produced by active secretion, ultrafiltration (20%), and osmosis (10%) by the pigmented epithelium of the ciliary body [25]. The rate of aqueous flow is approximately 2.75 µL/min with a turnover time of 1.5% per minute of the total anterior chamber volume. This turnover time might reduce the availability time of drugs to intraocular tissues, which might further be compounded by the binding of aqueous humour proteins to some drugs [21].

The major barrier to entry of systemic drugs into the posterior segment is the blood-retinal barrier. The retinal capillary endothelial cells and the retinal pigment epithelium (RPE) make up the inner and outer blood-retinal barrier, respectively. To preserve vision through its biomechanical function, the RPE efficiently restricts intercellular permeation due to the tight junctions [20].

The vascular space of the choroid may adequately be reached by drugs delivered via the oral route due to the fenestrated choriocapillaries. However, the outer retinal-blood barrier restricts further entry. The situation is compounded by the anatomical and physiological features that have to be overcome if a drug has to be concentrated in the vitreous. The vitreous humour is a transparent gel-like fluid in the posterior segment of the eyeball. It aids in giving shape to the eye, is approximately 4 mL and contains mostly water (99.9%) with collagen fibrils, hyaluronic acid, and ions constituting the remaining 0.01% [25]. A potential space exists between the vitreous and the retina called the sub-hyaloid space. There is firm attachment of the vitreous humour and the anterior retinal layer at the ora serrata [18]. Other attachments include the optic nerve head, the macula, and the blood vessels of the retina. The aqueous humour in the anterior segment offers a gradient against which substances have to diffuse to reach the posterior segment. The binding of melanin in the iris to some drugs might further impede this diffusion by reducing the available drug. Owing to this, topically applied medications penetrate the vitreous poorly whilst systemic drugs may need to navigate both blood-ocular barriers [24].

### 2.3. Periocular and Intravitreal Administration

To overcome the challenges with topical and systemic routes of drug delivery, especially for the posterior segment, invasive methods may be employed [20].

The posterior segment may be reached via the trans-scleral route, or into the choroid, thus the systemic route, or through the tear film, cornea, aqueous humor, and vitreous. The conjunctiva has an epithelial barrier which limits the rate of permeation of substances. With the transcleral route, this barrier is bypassed. The drainage system of the conjunctival blood circulation may also reduce bioavailability of drugs by draining most of the drug back into circulation, thus negatively affecting vitreous drug levels [19].

The sclera is relatively permeable than the cornea; therefore, drugs administered periocularly like in posterior subtenon injections might penetrate the sclera and choroid and reach the neural retina and the photoreceptors.

Invasive trans-scleral routes via the pars planar into the vitreous (intra-vitreal routes) ensure a more efficacious delivery of drugs into the vitreous chamber. These delivery methods have revolutionized management of conditions like diabetic retinopathy where intra-vitreal anti-vascular endothelial growth factors are administered [23].

## 3. Ocular Pathology

The ocular surface continually gets exposed to environmental factors that might lead to inflammation. Left unchecked, the inflammatory process would fundamentally alter the architecture and function of the affected ocular structure thus negatively affecting normal vision [26]. In the cornea scars might ensue whilst in the retina, proliferative vitreoretinopathy may occur. Thus, there should be a regulatory mechanism creating an immunological balance that helps reduce inflammatory responses and limit microbial growth on the ocular source to preserve vision. Most of this regulation is enhanced by the immune privileges of the eye [20].

There is minimal interaction between the eye and the body, with most of the interaction being through the eye associated lymphoid tissue (EALT) [27]. This lymphoid tissue is mostly in the conjunctiva and is a form of mucosal associated lymphoid tissue (MALT) [28] The ocular mucosal immune system also includes the tear associated lymphoid tissue and the nasopharyngeal associated lymphoid tissue. The anterior chamber environment inhibits scarring in tissues like the iris. The lens epithelium on the anterior capsule may undergo metaplasia as a response to injury if not eventually rendering the lens cataractous [27].

### 3.1. Ocular Immunology

There are two types of antigen presenting cells (APCs) in the eye, viz. the bone marrow derived dendritic cells expressing MHC class II and the tissue macrophages [27]. There are no APCs in the central cornea whilst the iris and ciliary body has in the sub-epithelium and interepithelial tissue, respectively [26]. The APCs in the inner eye are unable to activate T-cells, thus there is inability to activate the delayed hypersensitivity reaction [27].

The aqueous humour has immunosuppressive properties and consists of Transforming Growth Factor Beta (TGFβ) which plays a critical role in suppression of Tcell proliferation and γ-interferon production [25]. If an antigen is presented into the anterior chamber, there is activation of an immunological response called Anterior Chamber Associated Deviation (ACAID) [28].

Response to injury within an eye is through an inflammatory process which may be followed by vascularization and scarring. The cornea has five layers, whilst the epithelium and Descemet membranes regenerate after injury; however, the bowman layer, stroma and endothelium do not regenerate, thus an injury to the Bowman layer and stroma may lead to scarring [18].

Gliosis might take place in nerve tissues of the eye. In the retina, this process might be in combination with Retinal Pigment Epithelium (RPE) proliferation. In the choroid the melanocytes do not proliferate in response to trauma and scarring is secondary to the fibroblastic activity of scleral fibroblasts [26].

Further, up-regulation of cytokines, growth factors and matrix metalloproteinases might lead to neovascularization in the cornea [28].

### 3.2. Anterior Segment Ocular Conditions

The transparent cornea is avascular and thus has no lymphatic drainage. Visual acuity is largely affected whenever there are changes in the corneal vascularity or hydration. It is important to note that the cornea interacts with the MALT through the limbal vessels which are at the junction of the cornea and the sclera. The cornea is highly innervated through the branches of the ophthalmic nerve. When there is paresthesia due to disease, healing might be compromised [26,29].

Diseases affecting the cornea might include ulcerative and non-ulcerative keratitis. This might be infectious or non-infectious. The modalities of treating corneal pathologies should take into consideration these differences [19]. Treating an infectious process with steroids as opposed to appropriate antibiotics might lead to deleterious effects. It should, however, be noted that the choice of a drug in treating keratitis should consider the drug-cornea contact which should be long enough to offer therapeutic effects of the drug [20]. Topical eye drops might not have enough contact time before they are cleared through the lacrimal pathway, whilst ointment and gels might sufficiently have longer contact time. Topical drops do offer the advantages of not altering much the visual acuity when compared to ointments and gels. Nevertheless, to maximize on contact time, the clinician might need to increase the frequency of administration of topical eye drops [19].

The ocular surface is kept moist by tears whose components are produced by the conjunctiva, lacrimal gland, and the eyelids [21]. The tears also offer growth factors, antimicrobial peptides, and immunoglobulins to the ocular surface [25]. The tears further aid in mechanical washing of foreign particles during autonomic blink reflexes. There is an additional mechanical barrier that the mucin layer offers through a glycocalyx covering over the cornea [19]. Diseases affecting the conjunctiva or eyelids might affect tear film composition. This might lead to dry eye syndrome. To mitigate this, punctual occlusion might be employed. Excipients such as carboxylmethylcellulose sodium can moisturize the ocular surface. If used as an eye drop, there is the challenge of reduced contact time with the ocular surface which makes ointments and gels advantageous for this role.

Some posterior segment conditions like glaucoma might benefit from topically applied medication. This is because the only modifiable risk factor for glaucoma medically is the intraocular pressure (IOP) which is influenced by aqueous humour production or drainage. Antiglaucoma agents have to penetrate the cornea or conjunctiva and sclera to have their therapeutic effects on various receptors intra-ocularly [30].

The ocular commensals also play a significant role in the immune system of the ocular surface. They contribute to immune regulation and IgA secretion by the ocular associated lymphoid tissue [28].

### 3.3. Posterior Segment Ocular Conditions

The proteases from microbials may disrupt the tight extracellular matrix thus rendering the eye prone to permeation of unwanted substances [26,29]. In retinal tissue, inflammation might lead to leaky blood vessels causing oedema and bleeding within the retinal tissue or into the vitreous [16].

To treat the conditions of the posterior segment such as retinal proliferative disease, age-related macular degeneration, or indeed chorioretinitis, drugs have to be efficaciously delivered into the vitreous gel [20]. Most of the topical drugs would not have therapeutic effects by the time they reach the vitreous due to the various barriers to be encountered [19]. Thus, for posterior segment disease, the drug has to be delivered directly into the vitreous via the pars planna as a trans-scleral injection or be delivered into the system via the intravenous route or orally. It is only the trans-scleral route that offers therapeutic concentrations of drugs into the posterior segment; the systemic route has to overcome the blood ocular barrier. [31].

The eye indeed has a dilemma of responding to injury like any other body tissue, but this response has to be balanced with preservation of its function. Although the following section is divided by subheadings, it should provide a concise and precise description of the experimental results, their interpretation, as well as the experimental conclusions that can be drawn from the use of stimuli-responsive DDS in ocular therapeutics.

## 4. Non-Stimuli-Responsive Nanomaterial Based Ocular Drug Delivery

Nanomaterials have long been used as tools to enhance the outcomes associated with conventional drug delivery systems such as off-target delivery, toxic effects, and frequent and inconvenient dosing [32]. The nanomaterials described herein generally have dimensions less than 1000 nm [32] and are made up of biomaterials that are safe and proven [33].

**Figure 3:**
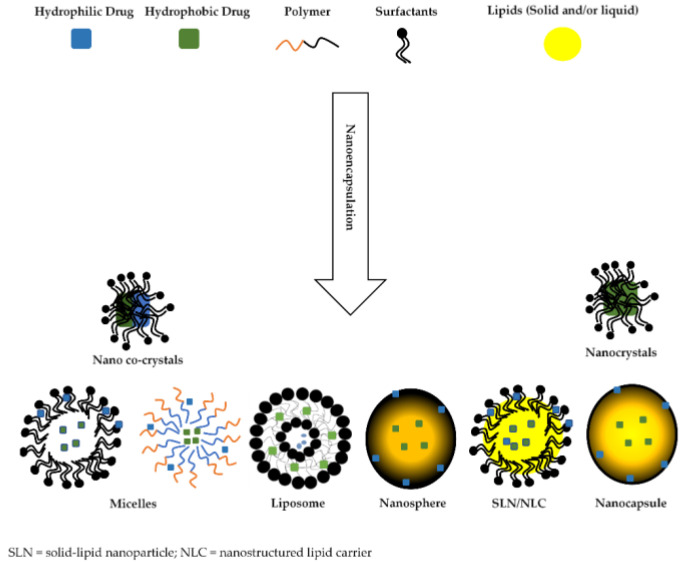
Summary of commonly developed and utilized biocompatible nanomaterials in ocular drug delivery. These nanocarriers possess the ability to accommodate and deliver both hydrophilic and hydrophobic drugs. [Reproduced from [33] and Nanomaterials MDPI in accordance with Creative Commons Attribution License (CC BY 4.0)].

A summary of the general nanomaterials used in ocular drug delivery and described herein is provided in Figure 3.

### 4.1. Polymer Based Ocular Drug Delivery Systems

#### Polymeric Nanoparticles

Nanoparticles are small particulate systems with a size range of between 10–1000 nm and carry a specific surface charge capable of enhancing their retention at a specific site [34]. The interaction of nanoparticles with the negatively charged corneal and conjunctival surfaces provides extended particulate retention and improves drug-corneal interaction [34,35,36]. Additionally, the use of small sized particles seeks to improve tolerance and adherence owing to their non-irritating characteristics, and possess the potential to provide sustained drug delivery, thus avoiding the need for frequent ocular drug administration [11,37]. The advancement in nanocarrier development has become increasingly important because of an additional advantage of scale-up feasibility [34].

Most nano systems are mainly made from polymeric and/or lipid materials, of both natural or synthetic origin, and are carefully designed to overcome ocular barriers [35,38]. They can deliver drugs to both the anterior and posterior segments of the eye, as they can efficiently cross the ocular blood retinal barrier [39,40]. Polymeric nanoparticles are generally classified as nanospheres and nanocapsules [35]. Nanospheres differ from nanocapsules in that they consist of drug dispersed evenly in a matrix system or adsorbed on the surface [35], while nanocapsules consist of a hydrophobic cavity surrounded by a thin polymeric membrane, wherein drug loading is achieved by surface adsorption and dissolving it in the particle nucleus as depicted in Figure 3 [35,41,42].

Polymeric nanoparticles show promise in enhancing solubility of lipophilic drugs; however, they still suffer some drawbacks; particularly, following intravitreal administration, they have been observed to aggregate, and very few in vivo studies of these delivery systems have been successful, although most pharmacokinetic studies have only been performed in animal models using rabbits [43].

Additional challenges with polymeric nanoparticle-based ocular DDS stem from multiple effects such as changing particle size, surface charge, and routes of administration [38]. Animal models have been utilized to test for ocular biodistribution of nanoparticles. Notwithstanding, the biodistribution data provides some understanding of nanoparticle distribution through ocular tissue although it has limited accuracy due to the limited number of animals used during the studies [38].

Timolol maleate (TML) is a non-selective beta-blocker and an agent of choice that has been used for more than 30 years to treat glaucoma. TML lowers intra-ocular pressure (IOP) by decreasing the formation of aqueous humour in the eye. However useful, TML suffers from some pharmacodynamic and pharmacokinetic drawbacks i.e., it has a short half-life (short acting), hence it requires multiple daily dosing of up to six times for extended therapeutic effect. Furthermore, commercially available TML eyedrops are rapidly eliminated from the corneal surface following administration, due to various ocular mechanisms actively removing the drug from the corneal surface, thus leading to reduced resident time and bioavailability [44].

Mittal et al. [44] investigated the use of polymeric nanoparticles for ocular drug delivery by using the ionic gelation method to prepare bioadhesive polymeric nanoparticles of flax seed gum (FX) and chitosan (CH) loaded with TML to treat glaucoma. In vitro studies were conducted, and the results demonstrated sustained TML release from the nanoparticles, whilst ex vivo studies for transcorneal penetration exhibited increased corneal permeation of TML when compared to commercially available TML eyedrops. Confocal scanning laser microscopy (CSLM) demonstrated the ability of TML to further penetrate to the deeper corneal layers. The nanoparticles were also found to be biocompatible during histopathological investigations. Additionally, animal model studies using rabbits showed that the nanoparticles successfully lowered the IOP in rabbits for extended periods compared to TML eyedrops. Therefore, these studies demonstrated great potential for successful and efficient management of glaucoma using bioadhesive nanoparticles [44].

In another study by Zhou et al. [45], lipid-polymer nanoparticles (LPNs) composed of poly poly (lactic-co-glycolic) acid (PLGA) nanocores containing a second-generation carbonate anhydrase inhibitor drug known as Brinzolamide (Brz) were prepared. Brz is known to have a significant effect on IOP levels, hence the drug is widely used to treat glaucoma and to reduce abnormally high levels of IOP. The aim of the study was to encapsulate Brz in a core-shell of LNPs and use them as carriers for the drug to evaluate the potential for LPNs to improve drug corneal permeation, prolonged release, and lowering of high IOP levels. Results from drug release studies demonstrated sustained release of Brz from the LPNs when compared to release from a commercial formulation of Brz eyedrops. Furthermore, in vivo studies showed that Brz-LNPs were successful in effectively reducing IOP, whilst maintaining a more prolonged therapeutic effect compared to commercially available Brz eye drops. From the in vitro studies performed using white New Zealand rabbits, it was demonstrated that Brz-LNPs exhibited enhanced corneal accumulative permeability compared to using eye drops. Improved corneal penetration of ophthalmic drugs is necessary to attain enhanced drug bioavailability. Data collected from these studies supports the use of Brz-LNPs; they show promising attributes of Brz-LPNs that warrant further investigations for their use as DDS to successfully treat glaucoma [45].

In an attempt to improve ocular residence time of antibacterial agents, de Vries et al. [46] used non-toxic lipid-modified DNA strands to form uniform micelles. In a single self-assembly process, RNA and DNA aptamers were hybridized with neomycin B and kanamycin B, respectively. The resultant functionalized nanocarriers had a good safety profile against primary corneal epithelial cells, adhered to human corneal tissue and that of living animals for hours thus showing improved residence time in comparison to pure drug solution, and had better antibacterial efficacy than pure drug on infected corneas. The authors envisaged that the findings of their study pave way to numerous possibilities for application of DNA-based delivery systems in ophthalmic use [46].

### 4.2. Lipid Based Ocular Drug Delivery Systems

#### 4.2.1. Solid Lipid Nanoparticles (SLNs) and Nanostructured Lipid Carriers (NLCs)

When used as excipients, lipids can accommodate lipophilic drugs where other drug carriers fall short. They can improve water solubility of different drugs, making them ideal excipients for use as drug delivery vehicles. Two main types of lipid-based nanoparticles are SLNs and NLCs [47]. Following their first patenting in 1996, SLNs have since been widely used by scientists due to their stability, non-toxicity, and reliability in their role as vehicles for drug delivery [48]. SLNs exhibit a spherical shape with a diameter size range of 50 to 1000 nm and are mainly composed of lipids that remain solid at room temperature, emulsifiers, a therapeutic agent/drug, and enough of a solvent system. SLNs have revolutionized the research field on DDS as they combine characteristics from different colloidal carriers such as polymeric nanoparticles, liposomes, and microemulsions [49]. Hence, the development of SLNs aimed to overcome shortfalls seen with using the aforementioned colloidal carriers as they demonstrate improved physical stability, targeted drug delivery and good drug release profiles. Further modifications to SLNs to improve drug loading capacity and stability resulted in the development of NLCs [47]. NLCs have considerable benefits over SNLs, hence they hold a more distinguished potential for application in pharmaceuticals [50].

Makoni et al. [51] manufactured clarithromycin (CLA) loaded NLCs to evaluate their envisaged potential to effectively treat ocular non-tuberculous mycobacterial keratitis. Their potential to increase ocular residence time due their polyethylene glycol-imparted muco-adhesive properties, in vitro drug release, and cytotoxicity against HeLa cells was investigated. The results of this study showed that the lipidic carriers had muco-adhesive properties suggesting their potential to enhance precorneal residence time, in turn increasing bioavailability. Furthermore, sustained release of CLA from the carriers was postulated to accommodate reduced frequency in dosing [51].

Fungal keratitis (FK) is a serious ocular infection associated with significant visual impairment. Natamycin (NAT) is the only drug of choice approved by the Food and Drug Administration (FDA) to treat FK. However, NAT exhibits poor corneal penetration when used to treat deep FK, which negatively affects its therapeutic efficacy. Khames et al. [52] prepared NAT-SLNs to achieve prolonged drug release and improve penetration of the drug through the cornea. NAT-SLNs release studies were conducted in vitro using artificial tear fluid. The results showed sustained, prolonged, and controlled release of NAT from the SLNs when compared to free NAT. A rapid release rate was observed with free NAT, with > 90% drug release occurring within two hours, whilst > 85% NAT release from SLNs occurred in ~10 h. The small particle size and lipophilicity of the SNLs allowed for them to successfully cross the corneal epithelium during ex vivo studies, resulting in enhanced NAT corneal permeability. Moreover, drug absorption was increased along with NAT bioavailability in the eye. Furthermore, drug-loaded SNLs were found to be non-irritating and exhibited improved antifungal effects and less corneal cytotoxicity [52].

In another study, Agarwal et al. [53] prepared meloxicam loaded chitosan nanoparticles (MLX-CS-NPs) for the treatment of ocular inflammation and evaluated their effectiveness in a rabbit eye model and in vitro. Results obtained from in vitro release studies showed sustained drug release that extended over 72 h, with desirable flux and drug corneal penetration of the cornea and sclera of rabbits. Furthermore, MLX-CS-NPs and meloxicam eyedrops were administered to the animals in an in vivo study. The results showed that the use of MLX-CS-NPs as eye drop dispersions improved the anti-inflammatory activity of the drug without any ocular irritation when compared to conventional meloxicam eye drops. Therefore, these results suggest that the use of chitosan nanotechnology increased residence time due to the positive surface charge imparted by the polymer, and overall enhanced drug activity [53].

Varela-Fernández et al. [54] successfully prepared lactoferrin-loaded NLCs (Lf-NLCs) to investigate their potential to improve the treatment of keratoconus, a non-inflammatory, chronic, and degenerative corneal disease with early young adulthood onset. Compared to that of lactoferrin (Lf) buffered solution, in vitro drug release behaviour from Lf-NLCs was more controlled and prolonged. Toxicity studies using bovine cornea showed that Lf-NLCs had no corneal toxic effects when compared to control formulations. Ocular surface retention studies performed ex vivo demonstrated a higher mucoadhesion percentage with the Lf-NLCs when compared to the control solution ex vivo. In vivo, NLCs showed desirable mucoadhesion and improved corneal permeability following direct cellular uptake. Therefore, the objectives of the study to enhance the pharmacokinetics and pharmacodynamics properties of the drug were successfully met. The suitability of using NLC for ocular drug delivery was proven, the authors demonstrated successfully controlled Lf release and enhanced corneal permeability from NLCs. Further, these nanolipid delivery systems demonstrate versatility, hence they can be applied in the development of various topical dosage formulations for ophthalmic drug delivery due to their physicochemical properties [54,55].Lipid nanocapsules containing cyclosporine (CsA), an agent used for the management of dry eye, and the corresponding blank formulations were prepared by Eldesouky et al. [56]. The drug loaded nanocapsules were incorporated into a thermoresponsive and muco-adhesive gel based on poloxamer 407- and chitosan- associated fabrication, respectively. The study aimed to target ocular tissue and provide long-term CsA levels and maximum drug tolerability. Both in vitro and ex vivo assessments were done using bovine cornea, and in vivo studies were done in rabbits to assess the biodistribution and efficacy of the novel carrier in a dry eye model. Comparisons with commercially available CsA ophthalmic formulations were made, and the results showed that CsA loaded nanocapsules in the gelling and mucoadhesive system could be a promising and effective ophthalmic formulation that is also non-irritating for treatment of dry eye. Furthermore, due to the increase in muco-adhesion of the gelling delivery system, increased residence time and prolonged drug release was observed [56].

#### 4.2.2. Nanomicelles

Nanomicellar carrier systems are composed of amphiphilic molecules that allow for the formulation of therapeutic drugs into clear solutions [57]. The lipophilic portion contains hydrophobic drugs, and the outer surface is hydrophilic and capable of increasing the solubility of drugs exhibiting poor aqueous solubility [10]. Micelles are either polymeric or surfactant in nature and can be encapsulated with efficiency, hence they are easy to formulate and can increase ocular drug bioavailability [6,57]. The increased interest in nanomicelles is because their small size allows for easy penetration of the lipophilic endothelial and epithelial corneal cells, as well as the hydrophilic corneal matrix [13]. This property overcomes solubility challenges seen with poorly soluble drugs; therefore, it enhances the penetration of the drug through the cornea and improves scleral spreadability and bioavailability on the anterior segment of the eye [10]. Nanomicelles have been suggested as the better option for the delivery of small drug molecules [13]. In addition, there have been studies suggesting the possibility of nanomicelle preparations effectively delivering drugs to the posterior segment of the eye. However, nanomicelles are not without limitations, that is, they become unstable overtime, have limited sustained release, and are not suitable for the delivery of hydrophilic drugs. Owing to this, these systems still need optimization and improvement for successful delivery of ophthalmic agents [13].

Li et al. [58] formulated an ophthalmic Soluplus^®^ micelle solution for effective ocular delivery of resveratrol (Sol-Res) for the promotion of corneal wound healing. Short- and long-term cytotoxicity test results showed no cellular toxicity (no eye irritation) and demonstrated enhanced cellular proliferation. Improved passive diffusion (in vitro) and corneal penetration in vivo of Sol-Res was observed, thus demonstrating the superiority of the micelle formulation over the free resveratrol solution which was used as the control in these studies. Furthermore, Sol-Res micelles showed improved chemical stability of the drug in aqueous solutions when compared to a free resveratrol solution [58].

Nikita et al. [59] prepared a topical nanomicellar formulation of everolimus, an immunosuppressant drug used in the treatment of posterior uveitis, and although desirable, treatment of this disease using the topical route cannot be achieved using traditional drug delivery systems. The aim of their study was to improve drug permeation through corneal epithelium with little to no ocular irritation, and to simultaneously enhance drug bioavailability in the posterior segment of the eye for the effective treatment of uveitis. The results of their investigations showed sustained release of everolimus from nanomicelles when compared to neat everolimus suspension. The everolimus nanomicelles had a higher permeability in goat cornea compared to everolimus suspension. These results suggest improved ophthalmic drug permeability and bioavailability, hence the administration of everolimus as nanomicelles can be an ideal therapeutic strategy in treating uveitis topically [59].

A study to improve solubility and stability of myricetin (Myr), a naturally occurring flavonoid with various pharmacological properties, demonstrated that developed Myr encapsulated polymeric micelles improved the aqueous solubility, stability, and corneal permeability of the molecule, thus enhancing its efficiency in ocular disease treatment [60]. From the results, it was observed that Myr polymeric micelles had a high encapsulation efficiency, as well as enhanced aqueous solubility and stability of the drug. Moreover, when compared to free Myr, cellular uptake and in vivo corneal permeation were improved in the micellar formulation. Furthermore, improved in vivo anti-inflammatory and antioxidant activity of Myr was observed compared to free Myr. Overall, these results suggest that micellar formulations of Myr could be an ideal delivery system effective in treating ocular pathologies [60].

Xu et al. [61] designed chitosan oligosaccharide-valylvaline-stearic acid (CSO-VV-SA) nanomicelles and hydrogenated castor oil-40/octoxynol-40 (HCO-40/OC-40) mixed nanomicelles according to Cequa™ (cyclosporine nanomicelle based eye drops for dry eye disease), recently approved by the FDA, in an attempt to improve posterior segment ophthalmic drug bioavailability. Dexamethasone (DEX), a potent corticosteroid used to treat macular edema due to its anti-inflammatory activity with a diminishing effect on vessel leakage associated with macula edema, was used as the model drug. Conventional topical ophthalmic formulations containing DEX fail to deliver the drug to the posterior segment of the eye, exhibit low corneal bioavailability, and require frequent dosing due to the short half-life of the drug, which can cause poor patient adherence and subsequently, therapeutic failure. Results from in vitro release studies demonstrated controlled drug release from the prepared nanomicelles. No rapid drug release was observed; the nanomicelles maintained effective concentrations and extended drug retention time on the corneal surface, hence excessive drug removal was avoided and ocular bioavailability to both the anterior and posterior segments was improved. Additional in vitro cellular studies to investigate potential cytotoxicity suggested that CSO and HCO-40/OC-40 nanomicelles were non-cytotoxic and had good biocompatibility, both of which are desirable attributes for ocular DDS. Furthermore, CSO-VV-SA nanomicelles and HCO-40/OC-40 mixed nanomicelle exhibited superior penetrating abilities compared to other nanocarriers found in literature. Altogether, topical eye drops containing these nanomicelles showed better retention abilities compared to conventional eye drops.

#### 4.2.3. Bilayered Lipid Based Drug Delivery Systems

##### Liposomes

Liposomal based formulations have been explored for the past decade as ocular DDS [62]. Liposomal structures are vesicular and consist of a lipid bilayer that surrounds an (in some instances more than one) aqueous phase, with a size range of between 10 nm–1 μm [62,63]. Liposomes are made from natural lipids and are therefore biocompatible and biodegradable [6].

Corneal drug absorption is increased with the use of liposomes; they are positively charged, and can bind with the negatively charged corneal epithelium or mucin to increase corneal residence time [6,63]. Furthermore, liposomal depots are inert and can thus provide extended drug release without altering the drugs intrinsic properties [6,64]. Liposomal preparations have shown compelling results from their use in intravitreal drug delivery [64]. They can also significantly increase the half-life of a drug, which offers attractive prospects in treating retinal disorders [63,64]. Nonetheless, deeper ocular tissue penetration is limited by their low binding affinity to ocular tissues. Overall, drugs administered using liposomes provide increased corneal permeability, in turn being efficacious at treating various ocular diseases [63].

Nanoliposomes have been widely used for their biocompatible and biodegradable characteristics and are applicable to ocular drug delivery due to their potential to increase both corneal permeability and retention time. Wang et al. [65] used nanoliposomes to encapsulate a poorly soluble drug Brz-hydropropyl-β-cyclodextrin (HP-β-CD) by inclusion complex (HP-β-CD/BRZ) to evaluate the potential for nanoliposmes to improve Brz local therapeutic efficacy. In vitro release studies showed controlled release of Brz, with enhanced penetration through the cornea along with extended therapeutic efficacy (lowering IOP). Moreover, safety tests conducted on New Zealand rabbits demonstrated a similar safety profile to that of the commercially available Brz ophthalmic formulation [65].

Wang et al. [66] identified the potential for corneal alkali burns to cause blindness due to oxidative stress. Ferroptosis, a form of programmed cell death reliant on iron and typified by lipid peroxide accumulation, was acknowledged as being the mediator of corneal injury due to alkali burns. Ferrorstatin-1, a ferroptosis inhibitor can effectively be used as therapy targeting ferroptosis; however, clinical application of this molecule is limited because of its poor aqueous solubility. Therefore, the authors developed ferrostatin-1-loaded liposomes (Fer-1-NPs) using soybean lecithin, cholesterol, and 1,2-distearoyl-sn-glycero-3-phosphoethanolamine-N-[methoxy (polyethylene glycol)-2000] for ferroptosis targeted therapy in corneal alkali burns management determined using in vitro assays, and in vivo studies in a mouse model. The results showed that Fer-1-NPs were effective in treating corneal opacity and neovascularization in vivo, and the treatment efficacy was comparable to that of DEX but without any undesirable adverse effects. In addition, the use of Fer-1-NPs showed no cellular toxicity which further confirmed biocompatibility of this novel delivery system. Therefore, Fer-1-NPs were identified to be a good candidate for the effective and safe management of corneal alkali burns [66].

Besifloxacin topical suspension is approved for the treatment of bacterial conjunctivitis and is used as a polymeric muco-adhesive system for controlled release. However, its use is associated with poor bioavailability similar to various other topical ophthalmic therapies [67]. Almeida et al. [67] incorporated besifloxacin into liposomes with amine additives (positively charged using either spermine or stearylamine) to evaluate the effect of the imparted positive charge on ocular drug delivery. They hypothesized that the charge may enhance penetration efficiency for burst drug release as well as improve residence time, in turn drug permeation. Liposomal minimum inhibitory concentration and minimal bactericide concentration were lower than the ones for Besivance^®^, a commercially available besifloxacin formulation. However, both formulations showed a similar increase in permeability following the application of an electric current. Although the incorporation of a positive charge did not impart an added advantage, passive drug penetration characterization using a novel in vitro ocular model to simulate lacrimal flow showed that the liposomal formulations had higher permeation than the Besivance^®^ (the control). Overall, passive topical delivery of besifloxacin was improved using positively charged liposomes, which were thus concluded as delivery systems that could potentially advance the management of topical ocular disorders [67].

Lai et al. [63] developed a liposome system that simultaneously entrapped berberine hydrochloride (BBH) and chrysophanol (CHR) with the intent to treat age-related macular degeneration. The overall aim of the study was to effectively deliver these drugs to the posterior segment of the eye. BBH and CHR have antioxidative, antiangiogenic, and anti-inflammatory properties; however, they have poor thermal stability and bioavailability, hence their clinical applications are limited. Polyamidoamine dendrimer-coated and uncoated liposomes were prepared. Subsequently, cellular uptake, in vivo transcorneal penetration, ocular drug absorption and irritation were investigated. Results showed that each preparation penetrated the corneal endothelium, and formulations did not undergo tear mediated eliminations; furthermore, drug cellular uptake was significantly improved with the polyamidoamine dendrimer coated liposomes. Moreover, the coated liposomes had enhanced bio-adhesion on corneal epithelium of rabbit eye and following evaluation of their therapeutic efficacy in an in vitro pharmacodynamic study, these carriers demonstrated protective effects in retinal pigment of humans and rats that had undergone photooxidative injury to the retina. After administration of polyamidoamine dendrimer coated liposomes, no side effects were observed on the ocular surface in a rabbit eye model, hence the delivery system demonstrated significant potential in the treatment of ocular pathologies.

iRGD is a peptide with enhancing abilities, it facilitates penetration of anti-cancer agents into blood vessels forming tumours for subsequent improvement of diagnostic sensitivity and therapeutic efficacy [68]. Huang et al. [69] prepared liposomes decorated with iRGD aiming to benefit from the binding and internalization characteristics of iRGD peptide. The liposomes were then loaded with Brz to improve corneal residence time, a measure of success for topical drug delivery, and penetration. Results showed that iRGD liposomes had a 3-fold increase in retention time when compared to conventional Brz eye drops. Furthermore, in vivo studies using animal models showed that iRGD liposomes could successfully penetrate the cornea within 30 min and continued for up to 3 h. Therefore, modified iRGD liposomes efficiently attained improved retention time, extended drug release, and enhanced drug penetration. Other in vivo and in vitro studies demonstrated that the Brz loaded iRGD liposomal system exhibited a controlled-extended release profile, could be topically administered to treat posterior ocular diseases, successfully lowered IOP with no systemic effects, and was biocompatible.

##### Niosomes

Niosomes are vesicular systems composed of non-ionic surfactants functioning as drug carriers [70]. They are nano-sized carriers composed of a hydrophilic head group and alkyl hydrophobic groups (one/two) forming a vesicle capable of encapsulating both hydrophobic and hydrophilic molecules [71]. Niosomes have been identified as vehicles of choice in ophthalmic drug delivery as they offer many advantages over other vesicles, for example, liposomes. They present with lower toxicity, improved chemical stability, and enhanced drug availability at the target site. Further, the use of niosomes allows for improved therapeutic efficacy and the reduction of adverse effects [70,72]. Structurally, niosomes are comparable to liposomes as they both share similar physical qualities; however, niosomes are more advantageous due to their biodegradability and biocompatibility when compared to liposomes [71]. One majorly significant limitation of liposomes is their chemical instability when used as adjuvants. Liposomal phospholipids are susceptible to oxidative degradation when exposed to air; therefore, these phospholipids and the liposomes require purification, handling, and storage in an inert atmosphere such as nitrogen. Compared to the production of niosomes, the process of purifying naturally occurring phospholipids can be costly, as well as the synthesis of pure phospholipids. Hence, niosomes are preferable as they offer greater chemical stability and are cheaper to produce compared to phospholipid synthesis [72].

According to Baldino et al. [73], the successful production of niosomes is dependent on the type of surfactant and production method. Proposed conventional methods for niosomal production include thin-film hydration, solvent injection, reverse-phase evaporation, emulsion method, and freeze-drying. However, these methods are time consuming, require the use of large organic solvents, and offer limited control over the shape and size of vesicles obtained. Owing to this, the authors reported on the improved production of these carriers using supercritical CO_2_ (SC-CO_2_) assisted processes to produce spherical, non-coalescing, and stable nano-niosomes.

Xue et al. [74] prepared niosomes loaded with latanoprost, one of the drugs used in the treatment of glaucoma. Contact lenses have been identified as one of the best vehicles for ocular drug delivery for the achievement of effective glaucoma treatment. Drug loading methods such as contact lens (CL) soaking, nanoparticles, molecular imprinting, and supercritical fluids have been investigated. Thus far, these methods show promising results such as improving corneal resident time and exhibiting sustained drug release; however, they present with significant limitations such as the alteration of crucial CL properties. Therefore, the authors aimed to address the limitations seen with traditional CL soaking by using niosomes to enhance CL drug loading capacity and attain extended drug release. Latanoprost-laden niosomes were prepared, and following the fabrication of silicone contact lenses, they were then soaked in solution containing latanoprost-laden niosomes (SM-LT-N-CL) and free latanoprost solution (LT-SM-CL). The amount of drug release from SM-LT-N-CL and LT-SM-CL was evaluated using both in vitro and in vivo release studies. In vitro release studies showed a high initial burst release from LT-SM-CL whilst extended drug release (between 48–96 h) was observed from SM-LT-N-CL without alteration of critical CL properties. In vivo drug release studies using New Zealand white rabbits was compared to that of single drops instilled on the rabbit’s eyes using conventional latanoprost eye drops. The results from LT-SM-CL showed sustained drug release (up to 24 h) while those from SM-LT-N-CL were sustained up to 72 h. The eyedrops exhibited increased release during the first few minutes, a rapid decrease in drug concentration in the tear fluid, and the drug could not be detected after 6 h. These results showed a unique application of niosomes using contact lenses for enhanced ophthalmic drug delivery [74].

Gupta et al. [75] prepared a niosomal in situ gel loaded with Brz to achieve prolonged release and effective IOP lowering in rabbits following single administration. In vitro release studies from the noisome in situ gel formulation and noisome solution were compared to the release from pure drug solution. Both niosomal formulations exhibited extended-release profiles of up to 24 h with maximum drug release of 81.14 ± 1.2% from the noisome solution and 78.88 % from in situ gel. Drug release from plain solution was observed to last up to 6 h. Ex vivo release studies showed that both noisome formulations improved drug corneal permeation and bioavailability. The gel demonstrated a slow and controlled release pattern attesting to its mucoadhesive properties. The formation of a drug reservoir is responsible for the increased retention time and subsequently the sustained drug release. Additionally, the in-situ gel formulation showed improved and sustained IOP lowering effect when compared to commercially available Brz eye drops in rabbit eyes. Therefore, these results show promising prospects in the use of Brz-laded niosomal in-situ gel for effective IOP lowering and sustained therapeutic effects in glaucoma treatment [75].

#### 4.2.4. Nanoemulsions

Nanoemulsions are carriers composed of two layers (oil and water) separated by a surfactant, with a size range of between 10–1000 nm [36]. Nanoemulsion drug carriers can be classified into four different phases: oil phase, aqueous phase, surfactants, and co-surfactants [76]. They are thermodynamically stable, cost effective, and relatively easy to make [36,76]. Careful selection of the phase used during formulation along with surfactants/co-surfactants is essential, as these components can influence the toxicity and stability of the delivery system [76]. Nanoemulsion carriers have shown promising results for drug delivery to ocular tissue [57,76].

For instance, Tayen et al. [77] investigated the development of terbinafine hydrochloride (T-HCL) as an ion-sensitive nanoemulsion gelling systems using gellan gum solution. The T-HCL gelling system demonstrated zero-order kinetics during in vitro dissolution testing and increased bioavailability in a rabbit eye model. Furthermore, the gelling system showed lower ocular irritation than a control of oily-drug solution, and had a significant permeability coefficient (log P) plus acceptable stability when subjected to various environmental stress conditions. Therefore, for ocular drug delivery, oil in water emulsions is most preferred as they demonstrate minimal ocular irritation and more tolerance when compared to water-in-oil emulsions [77].

Fardous et al. [78] developed a nanogel emulsion using beeswax as an organogelator to potentially deliver hydrophobic drugs to the posterior segment of the eye for the potential treatment of posterior segment eye diseases (PSEDs). A gel-in-water (G/W) nanoemulsion was prepared, and in vivo corneal permeability of the nanoemulsion eye drop preparation was evaluated. Results obtained from in vivo studies suggested increased G/W retinal layer permeability without ocular irritation. Biocompatibility was also evaluated using rat hepatoctytes and human umbilical vein endothelial cells which were exposed to various concentrations of the nanoemulsion. From the generated data, the prepared G/W nanoemulsion was observed to be biocompatible and considered to be a potential scaffold for ocular drug delivery to the posterior chamber of the eye, thus improved therapeutic efficacy in the treatment of PSEDs.

Acyclovir (ACV) containing nanoemulsions were prepared by Mohammadi et al. [79] to evaluate their potential for ophthalmic delivery of the drug. Release studies were conducted, and results were compared to a control group. ACV nanoemulsions demonstrated a controlled drug release profile, and permeability studies showed enhanced bovine corneal penetration compared to that of the control group. Furthermore, the ACV nanoemulsions exhibited a good safety profile and were found to be suitable for use as ocular DDS [79].

Dukovski et al. [80] evaluated the use of stearylamine cationic nanoemulsions as delivery systems for hydrophobic drugs to improve their ophthalmic delivery. Addition of a non-ionic surfactant stabilized the nanoemulsions and allowed for tuning of stearylamine to obtain a desirable balance when interacting with mucin, and subsequent biocompatibility. In their investigations, muco-adhesion and biocompatibility were evaluated using in vitro studies with a T-cell based corneal epithelium model. Oil-in-water emulsions were prepared using different types of oils, and nanoemulsions without stearylamine were used as negative controls. Results suggested that the formulation with stearylamine was more stable compared to the one without stearylamine, and in vitro muco-adhesion was demonstrated through significant interaction of the test formulation and mucin. Therefore, oil-in-water emulsions could potentially be used to improve solubility of lipophilic drugs and enhance their ocular availability by improving corneal residence time.

Kassaee et al. [81] developed and evaluated the potential for besifloxacin-loaded nanoemuslions to provide controlled drug release and potentiate corneal permeability. Ex vivo studies for corneal permeation showed a 1.7-fold increase in drug corneal permeation when compared to the besifloxacin suspension. Moreover, the nanoemulsions showed good tolerability on the bovine eye with no ocular tissue damage. Although nanoemulsions were loaded with lower drug concentrations (0.2%), therapeutic efficacy of the nanoemulsions was comparable to that of the suspension containing higher concentrations of besifloxacin (0.6%). Overall, the use of nanoemulsions as vehicles for ocular drug delivery warrants consideration as alternatives to using the conventional besifloxacin suspension formulation for the treatment of ocular bacterial infections [81].

### 4.3. Pristine Nano Drug Delivery Systems

More than 40% of newly discovered drugs have poor aqueous solubility, and various approaches have been attempted to enhance their solubility, with nanosuspensions being identified as promising candidates to fulfil that purpose [82]. Nanosuspensions contain insoluble drug molecules in a nanosized heterogenous colloidal dispersion and surfactants are used to maintain stability of drug particles in the formulation [82,83]. Sustained release of drugs with low aqueous solubility is possible when using nanosuspensions as they can also increase drug retention in tissues. Further, nanosuspensions are ideally prepared for drug molecules of high molecular weight, high log P, and high melting points, which are factors affecting formulation development of these molecules [82].

Glucocorticoid ophthalmic nanosuspension formulations have successfully been prepared using drugs such as DEX, prednisone, and hydrocortisone for the treatment of inflammation [82]. Results showed extended drug absorption and increased ocular drug bioavailability which reduced the need for frequent drug administration when compared to conventional eye drop formulations [82]. Excipients such as cyclodextrins have been used to increase aqueous solubility of drugs and enhance their solubility [84]. However, cyclodextrin use in pharmaceutical preparations tend to increase formulation bulk [82]. Nanosuspensions alleviate this issue by maintaining the crystalline state of drugs, whilst allowing for their high drug loading during dosage form formulation [82]. The high drug amount is a major advantage for ophthalmic drug delivery, and nanosuspension formulations maintain low dose volume through the use of minimal harmful non-aqueous solvents and high pH [82,85]. Nanosuspensions for ophthalmic delivery offer additional advantages such as sterilization, less irritation, and ease of ocular administration [6].

Olapatadine hydrochloride (OLO) is an agent used in allergic ocular diseases, and a nanoparticle suspension containing this agent was developed by Guven and Yenimlez [86]. They aimed to improve corneal residence time following topical administration, thus improving ocular drug bioavailability. In vivo and in vitro studies were conducted to investigate drug release and retention time, respectively. Successful drug loading into nanoparticles was achieved and the obtained release profiles of OLO from the nanoparticulate suspension were compared to that of a pure drug solution. The results showed that approximately 90% of the drug was released in 48 h from the nanoparticle suspension, while the same amount was released from the drug solution in 3 h. From these findings, it is evident that nanoparticulate suspensions provided improved extended drug release. There was no significant difference in drug retention between that of the nanoparticle suspension and the drug solution; however, the pure drug was washed off within 24 h. Results from drug release studies suggest that the drug loaded nanoparticulate suspensions have the potential for use as DDS to reduce dosing frequency. Moreover, they can improve patient compliance by providing extended drug release and increased corneal retention in the treatment of ocular diseases.

Pignatello et al. [87] evaluated the effect of a nanosuspension of ibuprofen sodium salt by topical administration in rabbit eyes [82]. The results demonstrated enhanced penetration of the anterior segment with increased drug levels in the aqueous humour, along with prolonged drug release when compared to conventional eye drops [82,87].

Hanagandi et al. [88] developed and performed in vitro-ex vivo studies on a brimonidine tartrate nanosuspension contained within an in-situ gel. The prepared nanosuspension was compared to a commercially available eye drop formulation used in the treatment of glaucoma. Sustained in vitro drug release was observed for over 24 h and drug permeation was significantly increased compared to that of a commercially available aqueous drop formulation. Overall, the formulated nanosuspension contained in an in-situ gel base showed great potential as a DDS for the management of glaucoma with reduction is dosing frequency (once daily dosing).

## 5. Stimuli Responsive Ocular Drug Delivery Systems

Stimuli-based delivery systems function in response to environmental triggers or external stimuli mimicking biochemical processes, thus prompting various actions at a specific target site leading to subsequent drug release via different mechanisms. These DDS have undertaken a significant role in the field of nanotechnology as they exhibit controlled drug release at targeted sites of action. Targeted drug release using stimuli-responsive DDS is due to specific triggers i.e., exogenous and endogenous stimuli. Over the past few decades, different external stimuli have been reported, which can be used to control payload release in ocular applications such as magnetic field, light, electrical field, and ultrasound [89]. Designs based on these novel DDS are based hydrogels, polymeric materials, and nanoparticles [90].

Exogenous drug delivery systems have the potential advantage of circumventing inter-patient variability since the release of payload is controlled by precisely controllable external factors [89].

Stimuli-responsive based DDS must fulfil certain rudimentary principles to qualify them for use as ophthalmic drug carriers, such as having high encapsulation efficiency, good safety profile, biocompatible, and biodegradable. Further, they must aim to avoid or limit adverse effects and ocular irritation observed with the use of conventional eye preparations. Various ocular DDS make use of polymeric materials based on the many advantages they offer; they can absorb large amounts of water, maintain structural integrity in aqueous media, and are biocompatible. Different factors underpin the development of effective ocular stimuli-responsive DDS i.e., specific ocular pathology and route of administration, along with environmental stimuli effects surrounding site of lesion, after which specific modifications of existing stimuli-responsive polymers (SRPs) can be made to meet the ideal properties necessary to achieve optimal ophthalmic drug delivery. Summarized properties ideal for the development of ophthalmic stimuli-responsive DDS using polymers and modes for improvement of stated properties are presented in Table 1 [91].

### 5.1. Exogenous Stimuli

#### 5.1.1. Magnetic Field-Responsive Drug Delivery Systems

The use of magnetic fields is widely applicable in medical diagnosis via the application of magnetic resonance imaging (MRI) [92]. The use of magnetic fields is rather new in drug delivery but highly useful. There are generally two mechanisms of drug release associated with magnetic fields, viz. targeted drug delivery by use of an external magnetic field followed by release [89], and making use of the heat generated by the magnetic mediator to trigger the release of payload from the thermal responsive carriers. The second mechanism would be construed as a hybrid between thermos-responsiveness and magnetic field triggered release and is termed the magnetocaloric phenomenon [15].

The use of magnetic fields to modulate drug release has been attempted with success. Pirmoradi et al. [93] designed and manufactured a magnetically responsive microelectromechanical system (MEMS) device that responded to an exogenous magnetic stimuli triggering the release of docetaxel (DTX). This was developed in attempt to suppress the development of new abnormal blood vessels in proliferative diabetic retinopathy [93]. In these works, a magnetic membrane composite was developed by incorporating coated iron oxide nanoparticles into a polydimethylsiloxane (PDMS) matrix with subsequent sealing of the drug loaded micro-container with a magnetic PDMS film [93]. The drug release controlling mechanism followed application of an external magnetic field that would result in deformation of the PDMS membrane, and the payload would then be discharged from the laser-drilled aperture. The results from this work showed that there was a 64-fold higher drug release under external magnetic field when compared to the passive drug leakage in that absence of an external magnetic field [93]. The authors postulated that despite the drug delivery being controlled and on-demand by virtual of adjusting the magnetic flux and/or actuation cycles, the technology was more suitable for lipophilic payloads drugs [93].

Similarly, it has been proposed that an anti-vascular endothelial growth factor (VEGF) receptor-1 peptide loaded magnetically modulated polymeric implant could have great potential in treating retinal neovascularization diseases [94]). Wang et al. confirmed, through in vivo experiments, that under application of a magnetic field drug release was triggered by the micropump and could be delivered to the macular area of the eye of the rabbit a few hours post triggering [94]. In the absence of the trigger signal, drug diffusion could be avoided. The main advantage of these technologies is that they can be tailored to provided drug release according to patients’ health status during long-term treatment. However, the use of these technologies requires invasive procedures, which could subsequently increase their overall cost [94].

Wu et al. [91] designed slippery magnetic micro-propellers intended to specifically target the optic disc site [95]. Silicon dioxide was utilized as the structural element of the micro-propeller, while nickel or iron were used as the magnetic segments. For the reduction of surface adhesion to the vitreous matrix, a perfluorocarbon liquid layer was utilized. In the presence of the magnetic field, it was noted that the micro-propellers were capable of delivering payload at a rate < 8 µm/s and reach the optic disc from the center of the vitreous within half an hour, which was 10-fold faster than through passive diffusion. It could be concluded that the magnetic field mediated drug delivery technology could not only accurately reach the target sites, but could, additionally, develop into a prospective magnetically triggered drug release system in the future [95]. However, the safety of using the magnetic chain segment on the retina and optic nerve is yet to be established and requires consideration to prevent severe functional and/or pathological damage to the retina in the long-term [95].

It has been proposed that these magnetic field triggered and directed technologies could be further augmented with other nano-vehicles such as liposomes/niosomes and core-shell nanoparticles to remotely release payload in specific regions of the eye [96].

#### 5.1.2. Ultrasound-Responsive Drug Delivery Systems

For quite some time in medicine, ultrasonic energy has been utilized for diagnostic purposes with specific applications in imaging. However, the use of ultrasound has recently found use in therapeutics with specific emphasis on drug/gene delivery. Ultrasonic energy has been demonstrated to successfully induce drug release from both biodegradable and non-biodegradable matrices in in vivo and in vitro experiments [89].

Penetration of active pharmaceutical ingredients (API) into tissues can be easily managed by working on ultrasound exposure time, the frequency, and cycle of duty [19,20]. Ultrasound reactive nano-vehicles include microbubbles, nanobubbles, nanodroplets, polymeric micelles, microemulsions, and liposomes [97,98].

Ultrasonic energy triggered payload release can be achieved via two general mechanisms, viz. destruction of the drug-loaded carrier resulting in release the drug, or by demolishing the chemical bond between the drug and the carrier vehicle [97,99]. The drug-loaded carriers can be destroyed by ultrasound-induced cavitation or ultrasound converted thermal/mechanical effect [89,100,101,101,102]. Cavitation refers to the process in which the pressure in the liquid drops rapidly thus resulting in the formation of small cavities [101]. In the process of ultrasonic energy triggered payload release, the cavitation effect is more important than the thermal effect [99]. Generally, there are two distinct types of cavitation in the process of payload release, viz. transient cavitation and stable cavitation [99,101]. Stable cavitation is characterized by oscillation of bubbles under the action of low-frequency ultrasound [99]. During this process, the size of the bubble changes with its static or equilibrium state, and liquid micro-streams around the bubble begin to form. The formed micro-streams subsequently result in shearing and release of the encapsulated payload [99]. This stable cavitation is simultaneously complemented by an alternative important effect which is characterized by the appearance of reversible pores on the surface of capillaries and cell membranes, thereby enhancing the permeability of cell membranes [103]. When the intensity of the ultrasonic energy is high enough, the oscillation of the bubbles occurs in an unstable manner, expands beyond their critical size, and collapses into smaller bubbles. This phenomenon is called inertial cavitation [99]. The collapse of this kind of microbubbles will generate strong shock waves and microjets [103], which will rupture the carrier and release the payload, as well as perforate the membrane acoustically while producing a strong thermal effect [104]. In another mechanism, destruction of chemical bonds could result in imbalances between the hydrophilic and hydrophobic regions of the molecules, resulting in destruction of the carrier architecture and irreversible release of payload [99].

This approach has been utilized to deliver medicines to different parts of the eye successfully. For instance, Thakur et al. utilized and optimized the ultrasound-nanobubble strategy and assessed it against three retinal cell lines [105] as well as two independent ex vivo large mammal, viz. porcine and bovine eye models [106]. In an in vitro assessment involving retinal cell lines, the synergistic effect of nanobubbles and ultrasound and how they impacted each cell type was studied [105]. The in-formulation gas retention was observed as the clear driver for improving the outcomes of ultrasound-assisted delivery of a co-delivered macromolecule into the cells. This was only achieved with echogenic nanobubble formulations [105]. In the animal model study, it was observed that acoustic streaming was the primary driving factor for ultrasound-assisted intravitreal particle migration, with only gaseous particles being able to migrate under the assessed conditions. The studies conducted in the bovine eye models revealed that repeated cycles of corneal ultrasound have the potential to improve the migration of injected nanobubbles deeper into the posterior regions of the vitreous humour while simultaneously causing no observable acute damage to the ocular tissues. In the porcine eye studies, it was demonstrated that the orientation of the applied ultrasound impulse could control the direction of nanobubble migration. These results had significant bearing on the outcomes of potential treatment of diseases of the posterior eye glaucoma [106].

In a similar but in vitro model based study, Nabili et al. made use of ultrasound assisted drug delivery for tobramycin and DEX. Concerning the drug delivery of tobramycin, there was insignificant increase in permeability for ultrasound- and sham-treated groups [107]. Conversely, significant corneal permeability increases were observed for sodium fluorescein and DEX of 46%–126% and 32%–109%, respectively, achieved at all treatment parameter combinations when compared to sham treatments with the exception of the 1-MHz ultrasound applications for DEX [107]. The observed permeability increase was greatest at a frequency of 400 kHz and appeared to be greater at higher intensities applied. Further, histologic analysis showed structural changes that were limited to epithelial layers of cornea [107].

The same researchers further conducted an in vivo assessment in which there was a 2.8-fold and 2.4-fold increase in the DEX penetration through the cornea when using ultrasonic energy at 400 and 600 kHz, respectively [108]. However, minimal damage and structural changes were observed in the corneal epithelium of the ultrasound treated corneas [108]. From both studies, the authors do highlight additional studies are needed to fully investigate the safety aspects of ultrasound application in ocular drug delivery by monitoring long-term recovery of corneal barrier properties and safety of ultrasound exposure in different eye tissues including the lens and retina [108].

In another study, the possibility of delivering macromolecules transsclerally to the posterior segment using low-frequency and -intensity ultrasonic energy was investigated [109]. The transscleral barriers were observed to be restored a fortnight after ultrasound application. The retinal cells displayed intact electric response with no structural or morphological changes in the ocular tissues post-sonication [109]. The study demonstrated the potential for ultrasonic energy assisted drug delivery as a non-invasive approach for delivering payload transsclerally to the posterior segments of the eye [109].

Zhou et al. demonstrated that the use of an ultrasound-microbubble system could enhance the gene transfection efficiency of pigment epithelial derived factor (PEDF) and effectively inhibit the development of choroidal neovascularization (CNV). Following 4 weeks of treatment, the efficiency of the PEDF gene mediated by ultrasound-microbubbles was significantly higher than that of transfection mediation using liposomes [110].

Much in the same vein, Li et al. demonstrated that ultrasound -targeted microbubble destruction (UTMD) mediated transfection could consequently accelerate and improve targeted transgene expression in the retina [111].

A summary of the ultrasonic energy responsive drug delivery systems is provided in Table 2.

#### 5.1.3. Electrically Responsive Drug Delivery System

Making use of an electric current or field is a recent but very useful way of triggering drug release in general but even more so in ocular conditions. The use of polymers in this regard has proven to have remarkable outcomes in ocular drug delivery. Due to the fact that precise control of voltage is highly achievable, electrically triggered drug delivery systems can fine-tune drug release rate by changing electrical stimulation. Compared with other stimuli-responsive systems, electrical stimulation can achieve tighter control and adjustment of drug release [112].

Intrinsically conducting polymers (ICPs) are organic materials consisting of alternating single and double bonds, that enable the ICPs, much like metals, to exhibit good electrical, optical, and magnetic properties [113,114]. Furthermore, ICPs are easily processible and synthesizable making them ideal for electrically triggered drug delivery [113,115]. Much like the aforementioned exogenous stimuli triggered drug delivery systems, ICP-based drug delivery systems are capable of on-demand/continuous delivery of payload making use of a myriad of mechanisms such as carrier structure destruction, redox reactions, and secondary stimulation of the thermosensitive carriers by generation of heat via stimulation by electricity [89]. ICPs can be prepared either by electro polymerization or by chemical polymerization [116].

Due to retinal cells being able to respond to electrical stimulation, ICP-based materials are an attractive option for applications in ocular drug delivery [114].

In the present context, age-related macular degeneration (AMD) and diabetic macular oedema (DMO) are treated by frequent administration via intravitreal injections. In providing an alternative to the standard of care, Ramtin et al. [117] developed a novel dexamethasone sodium phosphate (DSP) loaded electrically-responsive implant by electro-polymerization for the treatment of AMD and DME. Polypyrrole (PPy) was electro-polymerized with DSP to prepare conventional films (CF) and ethanol rinse films (ERF). When compared with CFs, ERFs required an additional solvent washing step in the middle and end of the electro-polymerization reaction. In this way, it is possible to remove PPy oligomers from the polymer surface resulting in smoother film surfaces [117]. The result of this increased smoothness and reduced impedance makes the ERF films more sensitive to electronic triggers. In vitro payload release experiments demonstrated that post first electrical stimulation, the ERF displayed a greater burst release, with the cumulative drug release volume was ~65 μg after 48 h while that of the CF was ~40 μg. Due to the biocompatible nature of PPy, the drug delivery system showed no cytotoxicity in cell counting kit-8 assay on ARPE-19 cells.

In an attempt to maximize drug loading, Uppalapati et al. prepared PPy particles encapsulated with both dexamethasone and DSP making use of drug-loaded micelles as a soft template to guide pyrrole chemical polymerization [116]. It was observed that DSP exhibited superior sensitivity to electrical stimulation triggered drug release when compared to uncharged dexamethasone base, highlighting the importance of electrostatic force in payload release. Examination of different electrical stimuli on drug release was performed and it was observed that alternating potentials were the most effective stimuli. At the end of 3 h, the in vitro release indicated that DSP released at an alternating potential was ~3.4-fold more than the drug released in the absence of stimulation. Furthermore, it was observed that under oxidation stimulation (+0.6 V), there was ~1.9-fold greater drug release when compared to reduction stimulation (−0.6 V). Correspondingly, there was a significant difference in the release of DEX under alternating potential stimulation under oxidation and reduction stimulation (*p* < 0.001) [116]. Intermittent application of electrical stimulation at 24 and 48 h resulted in the amount of drug released during the second application of electrical stimulation being greater when compared to the first time. This is attributed to polymer volume expansion under the action of electrical stimulation, which in turn enhances the release rate of the drug [116].

In ocular applications, the main drawback in utilizing these materials is the lack of biodegradability necessitating invasive surgery from implant placement and removal. As such, development of biodegradable ocular implants remains a real opportunity. A possible solution suggested to circumvent this is to develop drug delivery systems that utilize ICPs in combination with biodegradable materials or more flexible polymers to achieve overall better performance [112,118].

#### 5.1.4. Light-Responsive Drug Delivery Systems

As a stimulus, light is non-invasive and readily available making light-triggered drug release systems in ocular conditions an attractive option.

In general, the light utilised in photo-responsive drug delivery can be categorised into three general groups based on wavelength, viz. ultraviolet (UV) (200–400 nm), visible (Vis) (400–700 nm), and near infrared (NIR) (700–1000 nm) lights [119]. Light with short wavelengths has higher energy than the ones with long wavelengths [120]. The most commonly used light source is UV as it can provide more energy but possess challenges with high retinal photo-toxicity [119,120,121,122].

Light-responsive drug release systems can minimise invasive procedures and can deliver payload to the posterior eye segment with minimal invasiveness. Compared to other stimuli such as pH, temperature, and ion-triggered in situ gel, light-responsive systems could possess faster sol–gel conversion and are capable of correctly forming the structure of the systems in terms of hardness and porosity, ensuring the integrity of the implants. Light responsive systems have the capability of prolonging the retention time of the implant in the vitreous while reducing the diffusion of the drug from the polymer matrix. This results in minimizing the toxic side effects of the payload. The mechanisms that trigger the release of payload from the technology can be classified in four general groups, viz. photolysis, photoisomerization, Photo-crosslinking/decrosslinking and photothermal mechanisms [15].

##### Photolytic Light Responsive Drug Delivery Systems

In recent years, strides have been made to develop drug delivery systems based on photo-cleavage with applications in ophthalmology. For instance, Wang et al. [123] proposed a novel light-responsive nanoparticles cell penetrating peptide (NP-CPP) strategy for CNV treatment. The payload was administered intravenously, and the NP-CPP was intended to accumulate at the site of disease i.e the choroid and was converted to tissue-targeting state after exposure to blue light (50 mW/cm^−2^, 1 min, 400 nm). When exposed to light, the caging group would be removed by bond cleavage. It was envisaged that a photo-targeted treatment regimen could deposit the payload in an ideal spatial location and minimize “off-target” effects in other healthy tissues [123]). Using a laser-induced CNV mouse model, mice were treated with NP-CPP-DOX+hν experienced a reduction in neovascularization of ~46.1% [123].

In another study, a light-sensitive nanoparticle depot platform containing ortho-nitrobenzyl (oNB) moiety and nintedanib that incorporated fluorescein diacetate to observe photo-responsive drug release was proposed [124]. It was observed that 10 weeks post vitreal implantation, the light-sensitive nanoparticles retained their ability to release payload and inhibit angiogenesis. After irradiation with UV (365 nm, 8 mW cm^−2^, 5 min), the animals that received light-sensitive nanoparticles showed smaller CNV areas than those that received poly (lactic-co-glycolic) acid-encapsulated nintedanib rats. Despite, the photosensitive nanoparticles undergoing hydrolysis, the polymer can be retained in the vitreous humour for 30 weeks without degradation and thus showing by far superior retention when compared to traditional treatment period of 4–8 weeks [124].

Traditionally used timolol eye drops contain 3 × 10^7^ times more per drop than the required concentration of β-adrenergic receptors (βARs) in glaucoma treatment. More than 80% of the administered timolol is drained to the whole body [125], resulting in adverse reactions such as cardiac and respiratory dysfunction, headache, and blurry vision [126]. To circumvent this, Mu et al. [127] developed contact lens that are triggered by natural daylight. The contact lenses contained dimethoxy-substituted 2-nitrobenzene caged groups that can release timolol to the surrounding fluid through passive exposures to daylight to decrease the IOP in patients with glaucoma. The mechanism of drug release is photo-cleavable caged cross-linker with timolol post exposure to light in 400–430 nm region. The timolol light triggered contact lenses could effectively inhibit βARs and reduced off-target effect and adverse effects of excessive drugs by using only 5.7% timolol in a single eye drop [127]. The natural light trigger can sufficiently release timolol in 10 h period to inhibit βARs in eyes. Furthermore, passive control on the payload release was achieved by the exposure to light, and in a preclinical mouse model, payload was released from the contact lenses and could effectively decrease the IOP in the mouse model [127].

##### Photo-Dimerization Light Responsive Drug Delivery Systems

Due to their biocompatibility, fewer by products and lack of requirement of a photosensitiser, photo-dimerization-based light-triggered drug delivery systems are generally more desirable [128].

Wells et al. [129] developed photo-responsive PEG-Anthracene grafted hyaluronan as a controlled-delivery biomaterial for delivery of various high molecular weight anti-VEGF drugs and low molecular weight non-steroidal anti-inflammatory drugs for the treatment of neovascular age-related macular degeneration (nAMD). The technology developed was based on a photo-crosslinked dimer anthracene and was synthesized by an anthracene group bound by a PEG branch along the hyaluronic acid (HA) main link [129]. The photo-sensitive technology was capable of cross-linking HA up dimerization of anthracene and de-crosslink HA when anthracene dimer was de-dimerized. The technology developed could be placed in the vitreous for ~45 days. By altering the wavelength, dose, and irradiation time of the ultraviolet, the technology can modulate the amount of payload release allowing it to be tailored to the needs of the patients’ health status or stop releasing according to the occurrence of adverse reactions [129].

##### Photo-Thermal Conversion Light Responsive Drug Delivery Systems

Gold nanoparticles (AuNPs) are some of the most popular nanomaterials for light-responsive drug release which are capable of performing photo-thermal conversion by varying their sizes and shapes [129,130].

Depending on their sizes and shapes, AuNPs can absorb light of different wavelengths and can, therefore, be selected for specific use [122]. Lajunen and co-workers developed thermo/pH-sensitive photo-activated liposomes containing AuNPs, capable of selectivity in the acidic compartment such as endosome and lysosome in cells post exposure to Vis and NIR [131].

In a similar study, Basuki et al., developed an agarose AuNP loaded hydrogel depot that was responsive to Vis and modulate light induced release of multiple biologics such as bevacizumab [132]. The AuNPs developed were capable of absorbing blue light and performing photo-thermal conversion resulting in an increase in local temperature of the hydrogel > 45.0 °C. The gel undergoes a gel-sol transition to diffuse the drug to surrounding environment. The release of payload follows an ON-OFF cycle with light irradiation and evacuation. The release rate of bevacizumab is not different with or without blue light irradiation in the absence of AuNPs. However, when irradiated with blue light, the hydrogel system containing 0.1 mg/mL AuNPs, the bevacizumab release rate is 3-fold higher than that in the absence of irradiation [132]. The DDS developed can modulate the release of biological macromolecules from the AuNPs/hydrogel-based protein depot, resulting in a reduction of ophthalmic complications and the frequency of intravitreal injection [132].

Much in the same way, AuNPs-loaded liposomes were investigated as potential photo-sensitive DDS [130]. The liposomes investigated encapsulated hydrophilic fluorescent probe calcein which was released from the liposomes upon conversion of light to heat resulted [130].

One of the most critical factors to consider in drug delivery to the posterior segment of the eye using light-responsive hydrogels is biodegradability. It had been hypothesized that an injectable implant to deliver peptide to the posterior of the eye that has been loaded with nanoparticles, is biodegradable, light-responsive, in-situ-forming would be ideal [133]. The in situ forming injectable implants (ISFIs) are light-triggered liquid that undergo a sol-gel transition post light irradiation. The biodegradable nature of this technology allows it to be removed without invasive surgery post use [133]. Bisht et al., further proposed a biodegradable light-responsive ISFIs containing poly (lactic-co-glycolic) acid nanoparticles to deliver peptides to the posterior segment of eyes [134].

Tyagi et al. proposed a light responsive polycaprolactone dimethacrylate (PCM) and hydroxyethyl methacrylate (HEMA) in situ gel system to deliver bevacizumab (BCZ) in a sustained manner to treat CNV [135]. In these experiments PCM was used to cross-link HEMA in the presence of 365 nm UV light and 2,2-dimethoxy-2-phenylacetophenone (DMPA) as a photo-initiator. In vitro data suggested BCZ release could be continuously maintained over 4 months in the photo-crosslinked gel while maintaining biological activity. The fundus camera image showed the cross-linked BCZ-loaded gel remained in the suprachoroidal space for 60 days and the fluorescent signal could still be detected, while the gel that was not cross-linked lost its fluorescent signal on the 5th day [135]. Furthermore, the quantitative data from fluorescence spectrometry demonstrated that only 34% of the fluorescently labelled BCZ in the cross-linked gel was lost in the first day, and slow release could achieve over following 60 days [135]. Conversely, the uncross-linked gel lost 85% of BCZ from the onset, and the fluorescence signal returned to the baseline level on the 5th day. While BCZ possesses good stability during gel preparation, retention and release and the gel has good biodegradability the photo-initiator used in this system degrades into benzoyl radicals that are capable of causing oxidative stress and result in eye toxicity [135].

Despite the many successes and large potential of light-triggered drug delivery systems, some considerations need to be made when developing these technologies viz., paying attention to UV and chromophore toxicity [122] and that the toxicity of un-crosslinked gel-forming polymers as well as free photo-initiators to cells must be considered when photo-crosslinking [135].

### 5.2. Endogenous Stimuli-Responsive Drug Delivery Systems

Stimuli of biochemical and chemical origin are generally termed endogenous and include redox-responsive, enzyme responsive, pH-responsive, and ionic ocular microenvironment responsive drug delivery systems. These drug delivery systems can trigger payload release by regulating the ocular microenvironment, over-expression of specific enzymes, antibody-antigen interaction, and recognition of host-guest moieties in the eye [89].

#### 5.2.1. Temperature-Responsive Drug Delivery Systems

The use of endogenous temperature as a stimulus for ocular drug delivery has been by far the most explored to date [136]. They possess the ability to be administered in liquid form at lower temperatures < 25 °C and undergo a sol-gel transition after reaching physiological eyeball temperature ~32 °C [137,138], due to increase in temperature [139,140]. Many hydrogels with thermosensitive properties near this temperature have been developed as ocular drug delivery systems [141], such as Pluronics^®^, Poly (N-isopropylacrylamide) (pNIPAAm) and cellulose derivatives [142,143]. Generally, thermoresponsive behaviours of polymers can be categorized as possessing an upper critical solution temperature (UCST) or a lower critical solution temperature (LCST) [89,144]. These thermosensitive gels usually contain hydrophilic or hydrophobic groups such as -CONH and alkyl groups. In polymers characterized by having a LCST, upon the temperature dropping below the LCST, a hydrogen bond is formed between the hydrophilic polymer and the continuous phase, resulting in the polymer to exist in a swelling state and forming the gel [144]. However, when the temperature is above the LCST, the hydrophobic force between the hydrophobic polymer segments dominates overcoming the hydrogen bond force and causes the collapse of the polymer network [144]. Conversely, polymers with UCST characteristics collapse when the temperature is lower than UCST, and the increase in temperature increases the hydrophilicity resulting in swelling of the drug carriers [144]. The temperature change near the UCST or LCST results in change of the solubility and polymer structure, resulting in the release of the encapsulated therapeutic agents [104].

PF-127 is a widely used thermoresponsive material in drug delivery. In ocular drug delivery, solutions containing PF-127 exhibit a sol-gel transition at temperatures > 30 °C allowing for installation of a liquid gel that solidifies at ocular temperature. Zeng et al., designed a timolol maleate loaded thermoresponsive hydrogel to treat glaucoma [145]. The pharmacokinetic study in aqueous humour demonstrated that the T_1/2_, T_max_ and mean residence time (MRT) of drug loaded thermoresponsive hydrogel were 1.85-fold, 1.28-fold and 1.60-fold higher than that of the timolol maleate eye drops, respectively [145]. The in vivo rabbit glaucoma model demonstrated after a 7-day treatment period, the effect of timolol maleate eye drops in reducing IOP fluctuated greatly and had a tendency to rebound, while drug loaded thermoresponsive hydrogel steadily and continuously reduced IOP, which significantly prolonged the ocular bioavailability of the payload [145].

In another study using Pluronic^®^ as the thermoresponsive vehicle it was demonstrated how the effect of oxytetracyline was enhanced when drug loaded gelatin-polyacrylic acid nanoparticles were combined with Pluronic^®^ based hydrogels [146]. The optimized technology displayed a sustained effect against keratitis with an antibacterial activity comparable to that of commercially available product [146]. pNIPAAM is a thermoresponsive polymer that undergoes polymer phase separation at increased temperature and reaches a LCST at ~33 °C [147]. At 32 °C linear pNIPAAM produces unstable hydrogels that substantially collapse with increasing temperature above the LCST. Hsiue et al. [148] developed a formulation to treat glaucoma by encapsulating epinephrine in pNIPAAm for controlled release. The formulation based on 10% cross-linked pNIPAAm nanoparticles exhibited an IOP reduction effect that is 8-fold greater than conventional eye drops, and could last for 32 h [148].

Bellotti et al. [140] enhanced the performance of pNIPAAm thermosensitive hydrogels for glaucoma treatment by modulating the PEG content and its molecular weight in an attempt to reduce the LCST of the ocular gel, and achieving rapid gelation post administration ensuring the sol-gel transition of the hydrogel even in cold weather. The in vitro drug release curve shows that the anti-glaucoma drug brimonidine tartrate can be released in a sustained manner over 28 days [140].

Xue et al. investigated the biological activity of anti-VEGFs during a 40-day release in a thermoresponsive polyurethane hydrogel delivery platform [149]. The technology is capable of adjusting protein release by modulation the PEG/PPG ratio. In vitro data suggested that when the formulation comprised of 4 parts PEG and 1 part PPG, the neovascularization rate of the treatment group loaded with BCZ or aflibercept (AFB) was reduced by 80% when compared with the control group [149]. The data obtained from the rabbit choroidal neovascularization in vivo model proved that the thermosensitive hydrogel loaded with BCZ or AFB could effectively decrease the leakage of choroidal neovascularization [149].

Jimenez et al., developed a microsphere/thermoresponsive gel to improve the ocular biodistribution of cysteamine, a reducing agent used for treatment of cystine crystals in cystinosis, with sustained release properties [150]. The pNIPAAm based thermoresponsive gel system demonstrated sustained release properties and maintained cysteamine presentation across 12 h from a single drop [150]. These data demonstrated desirable distribution of cysteamine to the eye following topical administration coupled with high drug uptake to the cornea and low systemic distribution [150].

#### 5.2.2. pH-Responsive Drug Delivery System

The pH of tear fluid is approximately 7.4 [15] which provides an attractive opportunity for stimuli-responsive drug delivery while being cognizant that deviation in this pH may result in irritation. Such irritations may stimulate the production of tears and blinking, reducing the bioavailability of drugs administered on the surface of the eye [151]. Ocular DDSs based on pH-responsive polymers has been developed as an effective approach for the treatment of chronic ocular pathologies such as glaucoma [91]. They enhance the bioavailability of drugs rendered poor as a result of administration of eye drops to the ocular surface [91]. pH-Responsive polymers, also referred to as polyelectrolytes, contain weakly acidic or basic groups that accept or donate protons in response to changes in the pH of the surrounding environment [152]. Polyelectrolytes of basic monomers behave as cationic polymers under acidic conditions, while those with acidic monomers behave as anionic polymers under basic conditions [152].

Drug release in response to changes in the pH of the surrounding physiological environment can be a result of two strategies. In the first, pH-responsive polymers with abundant ionic side groups on the main chain undergo ionization state changes of these groups resulting in sol–gel transitioning [15]. They undergo conformational and/or solubility changes in response to change in environmental pH [15]. While in the second, some polymers contain chemical bonds that are unstable in acidic environments, such as hydrazone, oxime or acetals [15], while others are use acid-labile crosslinking agents, such as 3,9-divinyl-2,4,8,10-tetraoxaspiro [5.5]-undecane [153]. Changes in pH value on surrounding media triggers the cleavage of the bonds disrupting the amphiphilic balance of the copolymers, leading to the breakdown or degradation of the self-assembled nanocarriers, and the subsequent release of the encapsulated drugs [15]. Polymers that have been used in ocular applications include polyacrylic acid (PAA), cellulose acetate phthalate, polycarbophils and chitosan [15].

Carbopol^®^ is a PAA polymer that undergoes sol-gel transition as the pH rises from 4.0 to 7.4 [154]. It remains in solution state in acidic media and converts into a low viscosity gel on exposure to alkaline pH values [154]. Carbopol (pKa value of 5.5) is associated with eye irritations and damage in high doses. To mitigate this, it is often used in low concentration or in combination with methylcellulose and its derivatives to reduce the concentration of Carbopol^®^ [15]. Hydroxypropyl methylcellulose (HPMC) is commonly used in combination with Carbopol^®^ to reduce its acidity, at the same time enhancing the viscosity of the Carbopol^®^ solution [155].

Kouchak et al., developed a pH-responsive dorzolamide HCl loaded in-situ gel containing equal amounts of Carbopol^®^ and HPMC for the treatment of ocular hypertension. The clinical outcomes in-vivo indicated reduced irritation and a significantly improved lowering of IOP and less systemic absorption, compared to marketed eye drops [156].

Another study by Zhu et al., proposed a novel pH-responsive inner-layer embedded contact lens for controlled release of diclofenac sodium. The contact lenses were made with Eudragit S100, a copolymer of methyl acrylate with pH-responsive release mechanism soluble in media with a pH value above 7 [157]. The contact lenses are stored in care solution of pH 6.8, during which there is almost no loss of drug. In vivo findings demonstrated improved drug bioavailability in tear fluid with the soaked contact lenses compared to an eye drop solution. Control in drug release was also improved as the API was still detectable in the contact lenses 24 h after administration, while it was not detectable 2 h after instillation of the eye drops. This novel inner-layer embedded contact lenses can develop into DDSs that can provide sustained drug delivery that can last for one day after a single administration [158].

pH-Responsive nanomaterials have also been developed for ocular drug delivery. They can be constructed from organic and inorganic materials including polymers, lipids (liposomes, nanoemulsions, and solid-lipid NPs), metallic, and ceramic NPs [104]. For instance, Liu et al. developed a curcumin (CUR)-loaded nanogel for ocular drug delivery using cationic nanostructured lipid carriers (CNLC) and a thermosensitive gelling agent. In-vitro drug release, corneal permeation, ocular irritation and preocular retention experiments were conducted together with the pharmacokinetic profile of the formulation in aqueous humor utilizing the microdialysis technique. The AUC of the nanogel was higher than that of a curcumin solution by 9.24-folds, indicating a significantly improved bioavailability [159].

Pandurangan et al., formulated an in-situ gel loaded with SLNs as nanocarriers for spatial drug delivery of voriconazole on the ocular surface. The in-situ gel demonstrated good stability and excellent zone of inhibition in the microbial assay of voriconazole [160].

Natamycin is a polyene antifungal commonly used in the treatment of fungal keratitis. Polyenes do not penetrate well through an intact corneal epithelium, as a result their pharmacological effects are short-lived [161]. Paradkar et al., developed a natamycin-loaded niosomal in-situ gel using Poloxamer 407 and HPMC K4M. The in-situ gelling formulation demonstrated improved corneal retention time due to the bioadhesive properties of the polymers used and displayed sustained drug release of up to 24 h. The formulation demonstrated better transcorneal permeation, translating to improved drug bioavailability [162].

Similarly, Shukr et al., showed enhanced drug release portfolio with a significantly higher C_max_, delayed T_max_ and increased bioavailability, and the formulation was found to be non-irritant [163]. They formulated a voriconazole-loaded niosomal in-situ gel for ocular inserts using Span 40 and Span 60 with Pluronic F127 and Pluronic L64.

In order to achieve prolonged corneal contact time of norfloxacin (NFX) for treatment of extra ocular diseases, a pH triggered nanoparticulate in-situ gelling system was designed for ocular delivery. NFX loaded nanocarriers were developed using chitosan as a matrix forming polymer, with sodium tripolyphosphate as the crosslinker. The in-situ gel exhibited superior performance over the marketed eye drops [164].

Another approach to improve ocular retention time of drugs was studied by Gupta et al., by designing nanoparticle laden in-situ gel containing levofloxacin encapsulated PLGA nanoparticles, added into a chitosan in-situ gel. Following static and dynamic gamma scintigraphy evaluation in the in-vivo models, the developed nanoparticle laden in situ gel formulation showed a slow clearance rate and an extended corneal retention time compared to marketed formulation and in-situ gel alone [165]. This technique was applied by same group of researchers with sparfloxacin and showed excellent sustained release of the nanoparticle in-situ gelling system containing [166].

Furthermore, the in-vitro release of ketoconazole poly(lactide-co-glycolide) nanoparticles from an alginate-chitosan in-situ gel displayed a sustained and greater drug release compared to free drug formulations [167]. In addition, the in-situ gelling formulation demonstrated a higher and sustained drug permeation transcorneally, while the nanoparticles showed improved antifungal activity in comparison to pure drug formulations [167]. Sayed et al. developed a biodegradable microsphere-loaded ion-activated in-situ gel of ofloxacin (OFX). The in-situ gelling system showed improved relative bioavailability by 11.7-fold, compared to marketed OFX eyedrops in-vivo [168]. Furthermore, the in-situ gel demonstrated sustained release over a prolonged period, indicating that the in-situ gel could avoid frequent instillations, which will in turn improve patient tolerability and compliance [168].

A pH-triggered gel containing baicalin for sustained ocular drug delivery using Carbopol 974P and HPMC E4M was developed. The in-situ gel displayed a better ability to keep baicalin stable and retain drug release than marketed baicalin eye drops to enhance the ocular bioavailability. Sustained drug release was noted for a period for over 8 h. Furthermore, the AUC and C_max_ values were found 6.1-fold and 3.6-fold greater than those of the control solution, respectively [169].

Kesarla et al., formulated an ophthalmic in-situ gel using the ion-sensitive polymer gellan gum with moxifloxacin nanoparticles-loaded. The in-situ gel underwent sol-gel transition immediately after instillation and formed a strong gel that continuously released the drug over a sustained period of 10–12 h [170]. The formulation demonstrated improved corneal contact time, reducing the frequency of instillations [170]. Similarly, a novel ion-sensitive ophthalmic nanoemulsion (NE) based in-situ gel containing terbinafine hydrochloride was developed. The in-situ NE gel showed a significantly higher C_max_, delayed T_max_, prolonged corneal residence time and enhanced ocular bioavailability [77].

#### 5.2.3. H_2_O_2_ Responsive Drug Delivery Systems

Ordinarily and at physiological conditions, reactive oxygen species (ROS) are essential cell signaling pathway aides contributing to normal bodily function [171,172]). There are many ocular pathologies associated with inflammatory reaction creating an excess of ROS, such as glaucoma [173], AMD [174], cataracts [175] and DED [176,177]. 

When compared to normal tissues, the levels of ROS at these diseased sites are increased [172,178]. As such, ROS can be used to trigger release as well as enhance payload accumulation in these ocular microenvironments significantly. The ROS family includes molecular species such as singlet oxygen (^1^O_2_), hydrogen peroxide (H_2_O_2_), peroxide (O_2_^-^), superoxide (O_2_•^-^) and hydroxyl radical (•OH) among others [172,179,180,181].

The release principles behind various ROS-responsive delivery vehicles can be summed up as ROS inducing changes in carrier solubility, resulting in carrier decomposition or ROS-induced carriers’ cleavage and, finally, ROS-induced carrier-drug linker cleavage [182]. Depending on the type of payload incorporated, ROS-responsive drug carriers can load drugs through electrostatic interactions, hydrophobic interactions or covalent bonds resulting in differently tailored payload release [182].

The use of ROS-responsive drug delivery systems for ocular applications has not been completely explored. However, some research has been performed into realising the potential of ROS-responsive drug delivery systems. For instance, a glycol chitosan nanoparticle-based formulation containing cerium oxide has been developed for the treatment of DED [183]. In the presence of ROS this cerium (Ce) in the technology is capable of reacting with the microenvironment by oxidizing Ce^3+^ to Ce^4+^ resulting in the elimination of ROS [172,183]. The novel technology developed exhibited no cytotoxic effects and enhancements on DED models by scavenging ROS, stabilizing the tear film, up-regulating superoxide dismutase (SOD), promoting and maintaining corneal and conjunctival cell growth and integrity [183]. 

A H_2_O_2_ -responsive contact lens as developed to promote sustained payload release [184]. The stimulus exploited by this technology is not pathophysiological but as a result of the conversion of mechanical energy to chemical energy [184]. The pressure produced by the movement of the eyelid causes the cleavage of Si-O-Si bonds in siloxane-hydrogel contact lenses and produces H_2_O_2_. The H_2_O_2_ produced is sufficient to trigger the drug delivery system. In the process of releasing oxidizing agents, contact lenses did not show burst effect. It should be noted, however, that these works did not make use of a model drug [184].

#### 5.2.4. Ion-Responsive Drug Delivery System

Due to the rich abundance of ionic species such as Na^+^, K^+^, Mg^2+^ and Ca^2+^ in tears, exposure of anionic polysaccharides can result in interaction with polymer causing conformational changes and the formation of a three-dimenison network structure [185], culminating in a sol–gel transition [186]. Gellan gum, κ-carrageenan, and alginate are the most commonly utilized polysaccharides for the preparation of ion-responsive in situ gels [187,188].

Wu and coworkers developed a Ca^2+^ co-ordinated dexamethasone supramolecular hydrogel through ionic cross-linking strategy to control non-infectious uveitis [189]. The results demonstrated that an ion-responsive hydrogel could significantly reduce ocular inflammation. The concentration of Ca^2+^ has the ability to influence the sol-gel transition behaviour of the supramolecular hydrogel and can modulate the release rate of the payload. Moreover, the supramolecular hydrogel had a high encapsulation of payload as well as carrier-free features. The supramolecular hydrogel displayed a linear relationship between treatment effect and drug dose on experimental autoimmune uveitis rats [189].

Ion-triggered payload release can also be combined with contact lenses to further enhance the outcomes of treatment. Zhu et al. [143] designed an ion-triggered inner-layer embedded on contact lens for the triggered release of betaxolol hydrochloride. The in vitro drug leakage from contact lenses was stable within 30 days of testing. Pharmacokinetic results in rabbit models demonstrated that the maximum drug concentration of eye suspension was 18.8 ± 7.9 μg/mL, and payload could not be detected in tears after 8 h [143]. In vivo experiments further demonstrated that payload release could be maintained from the inner-layer embedded contact lens for 168 h.

While gellan gum is an ideal drug delivery polymer with sensitivity to the presence of polycations, alginate has also found considerable use in ocular applications for the same reasons. Kesavan et al. [190] developed an ion-sensitive hydrogel prepared from gellan gum or sodium alginate and sodium methylcellulose sulphate (SMCS) to treat experimental bacterial keratitis. When compared to commercially available drugs, the gatifloxacin hydrogel preparation exhibited stronger adhesion, which effectively prolonged the retention time on the surface of the eye. On the 4th day after treatment commencement, significant differences in keratitis scores i.e mucus removal, eye irritation, tear secretion, and eyelid swelling were observed between the two groups [190].

Díaz-Tome and co-workers designed ion-activated in situ gels based on gellan gum, κ-carrageenan or hyaluronic acid for the treatment of fungal keratitis [191]. Compared with a solution of free drug, PET-CT showed that the residual amount of drug in two econazole hydrogel preparations was approximately 2-fold greater than free drug solution 2 h post administration [191]. T mean MRT of two hydrogel formulations were 71.08 ± 41.66 min and 72.37 ± 26.90 min respectively, while the MRT of the free drug group was only 29.9 ± 11.56 min [191].

#### 5.2.5. Enzyme-Responsive Drug Delivery Systems

The ocular microenvironment is a host to a myriad of enzymes under both physiological and pathological conditions, including matrix metalloproteinases (MMP) in tissues, hyaluronidase in the vitreous [129], lysozymes [192] and esterases [193] in tears. The expression of many of enzymes expression changes under pathological conditions including tumours and inflammations and can be exploited to trigger payload release and promote drug accumulation in target sites resulting in enhanced local concentration of payload to achieve optimized outcomes.

Enzyme-triggered drug delivery systems are based on enzyme-responsive polymers, which can release drugs only under the catalysis of enzymes [104]. There are two aspects to enzyme triggered drug release. The first results from drug release occurring due to the polymer within which the drug is encapsulated is a substrate of the enzyme in the ocular microenvironment resulting in its physical/chemical properties to change under the action of a specific enzyme [104]. The second aspect of enzyme-responsive drug release results changes in internal interactions of the within nanomaterials within which the payload is harboured which may cause instability of the nanocarriers and trigger payload release [104].

Kim et al. reported a nano-diamond (ND)-embedded contact lenses containing chitosan, a lysozyme-cleavable polysaccharide, which was designed to deliver timolol maleate (TM) to treat glaucoma for a long period of time [192]. The lysozymes present in tears are capable of hydrolysing the 1,4-β-glycosidic bonds present in chitosan [194]). The ND coated with polyethylenimine (PEI) cross-linked with chitosan to form nanogels, and the nanogels were subsequently incorporated into poly-HEMA materials to cast contact lenses. The nanogel lense technology was tested on release rate of TM against payload-soaked contact lenses and molecularly imprinted contact lenses. The payload-soaked contact lenses and molecular imprinting contact lenses achieved complete drug release within the first hour [192]. Conversely, the ND loaded nanogel exhibited near zero drug release in absence of exposure to the enzyme stimuli. After exposure to the stimulus, the cumulative release from the ND nanogel was ~9.41 µg at 24 h [192]. The contact lenses displayed desirable controlled release performance of payload. Despite the technology that was developed being capable of circumventing the drawbacks regarding sustained release of payload, it was deficient in delivering therapeutically relevant quantities of payload in this condition. It, therefore, remains a big challenge to optimize the technology with regards to quantities of payload delivery while maintaining the sustained release profile [192].

While lysozyme triggered drug delivery is very attractive, other researchers have exploited other enzymes to achieve enzyme responsive drug release. For instance, Grimaudo et al. proposed esterase-triggered Pluronic^®^ F-127/D-α-tocopheryl polyethylene glycol 1000 succinate (PF-127/TPGS 1000) mixed micelles for the delivery of cyclosporine to treat anterior eye diseases such as uveitis, dry eye disease (DED) and keratoconjunctivitis [193]. Pluronic^®^ F-127 and TPGS1000 are capable of increasing the solubility and corneal permeability of cyclosporine while prolonging its retention time on the ocular surface retention time [193]. The payload-controlled release was mediated by TPGS1000 hydrolysis. In the presence of esterase, the hydrolysis of TPGS1000 results in destruction of the mixed micelles thereby releasing the payload. Furthermore, In the presence of esterase, TPGS1000 is metabolized into vitamin E and vitamin E succinate which are antioxidants with added beneficial effect on ocular pathology providing support to the therapeutic activity of cyclosporine [193].

A phenomenon known as enzyme-instructed self-assembly (EISA) has drawn considerable attention in the field of ocular drug delivery [195,196,197]. Self-assembly of small drug molecules, in vivo, provides an excellent opportunity for targeted and long-term accumulation of payload at the lesion site. Hu Et al., exploited this by designing an ibuprofen based phosphorylated peptide-drug (IBF-HYD-GFFpY) precursor utilizing an ester bond to release payload at the target site [197]. This was explored while also designing in vivo self-assembly to be achieved by the catalysis of alkaline phosphatase (ALP) in the tear fluid for efficient ocular drug delivery [197]. In vitro, the enzymatic experiments indicated that the dephosphorylation of IBF-HYD-GFFpY occurred firstly with the yield of IBF-HYD-GFFY which subsequently self-assembled into the supramolecular hydrogel to afford sustained payload release over 4 days. The novel technology exhibited superior anti-inflammatory efficacy compared to free ibuprofen (IBF) at the concentration of 200 μM when tested in a lipopolysaccharide (LPS)-activated Raw 264.7 macrophages model. Furthermore, the aqueous solution of novel technology administered via topical instillation exhibited near zero ocular irritation and displayed longer precorneal retention compared to the conventional eye drop formulation. Finally, in the in vivo assessment in a rabbit model of endotoxin-induced uveitis (EIU), the technology displayed comparable therapeutic efficacy of with that of clinically used 0.1% w/w diclofenac (DIC) sodium eye drops by the reduction of macrophage and leukocyte influx [197].

## 6. Future Perspectives and Conclusions

The use of the visual sensory aspects has a great effect on the quality-of-life individuals experience. Unfortunately, the eyes, much like other organs of the body, are susceptible to pathological conditions that require specialized and highly specific drug delivery. The use of nanotechnology and/or stimuli responsive drug delivery has been demonstrated to be capable of achieving superior drug delivery outcomes in treating many diseases of the eye. It appears highly beneficial for future outcomes in improving future outcomes to utilize nanotechnology and stimuli-responsive polymeric carriers to increase the therapeutic efficiency of the technologies. It remains highly imperative to make enlightened decisions in choosing the stimuli to exploit while keeping in mind the potential effect of the combination of a nanocarrier to target this ocular microenvironment to develop drug delivery systems capable of on-target drug delivery suited to the patient’s needs.

## Figures and Tables

**Table 1:** Summarized properties ideal for the development of ophthalmic stimuli-responsive DDS using polymers and modes for improvement of stated properties [91].

Ideal Properties of Ophthalmic Stimuli-Responsive DDS	Techniques for Improvement of Properties
Stimuli response	Confirm the stability of certain ocular environmental factors, i.e., pH, temperature, and ions observed in the diseased eye to ensure desirable in vivo response of intentionally used SRPs
Ocular biocompatibility	Select SRPs compatible with the ocular environment and ocular cells/tissues of interest in both solution and gel phases
Ocular biodegradability	SRPs chosen must be biodegradable in the ocular microenvironment to circumvent any blockage of the normal flow of the ocular fluid and development of any infections. If the SRPs are non-biodegradable, additional biodegradable biopolymers can be incorporated to improve the safe use of the resultant carriers. Further, selected SRPs must be inert to ocular metabolic activities.
Drug encapsulation and release	SRP modification can be undertaken to regulate response to a stimulus, such as employing a synergistic combination with other polymers to achieve immediate or sustained drug release.
Modifiable structure	The chemical structure of selected SRPs must allow for functionalization through the addition of specific chemicals or polymers with the potential to enhance the ocular delivery and therapeutic outcomes. The functional biomaterials should exhibit enhanced encapsulation efficiency of ophthalmic drugs, resist dynamic ocular fluids, while maintaining strong muco-adhesive performance, to potentially provide extra-therapeutic benefits unattainable with conventional ocular delivery systems.

**Table 2:** Summary of ultrasonic energy responsive drug release systems in vivo/vitro.

Ultrasound-Responsive System	Ultrasound-Responsive Release Mechanism	Therapeutic Agents	Irradiation Protocol	Ref
**Rabbit IgG antibody coated Nanobubbles**	Ultrasound induced sonoporation and permeabilization	Rabbit IgG antibodies	1 MHz, 1 W cm^−2^, 20 s or 0.5 W cm^−2^, 30 s	[105]
**Rhodamine-tagged gas entrapped nanobubbles**	Ultrasound enhances permeability of the cell membrane	-	1 MHz, 0–2.5 W cm^−2^, 50–100% duty, 60 s	[106]
**Free anti-inflammatory drugs, hormones and hydrophilic drugs**	Ultrasound enhancement of *trans*-corneal drug delivery via streaming	Tobramycin, dexamethasone and sodium fluorescein	400 KHz, 0.3–1.0 W cm^−2^, 5 min	[107]
**70 kDa dextran**	Ultrasound enhanced the permeability of macromolecules	Fluorescent dextran	0.12 W cm^−2^, 40 Hz, 90 s	[109]
**Mixture of plasmid and microbubbles**	Ultrasound promotes target plasmid entering into cell nucleus	PEDF gene	300 KHz, 0.5 W cm^−2^, 60 s, duty cycle, 20%	[110]
**UTMD**	UTMD intensify the bioeffect of sonoporation.	EGFP gene	1 MHz, 2 W cm^-2^, 60/120 s, 50% duty cycle, with the ratio of MBs to cells as 50:1	[111]

## Data Availability

Not applicable.
